# Molecular Mechanisms of Plant Trichome Development

**DOI:** 10.3389/fpls.2022.910228

**Published:** 2022-06-01

**Authors:** Guoliang Han, Yuxia Li, Zongran Yang, Chengfeng Wang, Yuanyuan Zhang, Baoshan Wang

**Affiliations:** ^1^Shandong Provincial Key Laboratory of Plant Stress Research, College of Life Sciences, Shandong Normal University, Jinan, China; ^2^Dongying Institute, Shandong Normal University, Dongying, China

**Keywords:** plant, epidermal cell, trichome, development, molecular mechanism

## Abstract

Plant trichomes, protrusions formed from specialized aboveground epidermal cells, provide protection against various biotic and abiotic stresses. Trichomes can be unicellular, bicellular or multicellular, with multiple branches or no branches at all. Unicellular trichomes are generally not secretory, whereas multicellular trichomes include both secretory and non-secretory hairs. The secretory trichomes release secondary metabolites such as artemisinin, which is valuable as an antimalarial agent. Cotton trichomes, also known as cotton fibers, are an important natural product for the textile industry. In recent years, much progress has been made in unraveling the molecular mechanisms of trichome formation in *Arabidopsis thaliana*, *Gossypium hirsutum*, *Oryza sativa*, *Cucumis sativus*, *Solanum lycopersicum*, *Nicotiana tabacum*, and *Artemisia annua*. Here, we review current knowledge of the molecular mechanisms underlying fate determination and initiation, elongation, and maturation of unicellular, bicellular and multicellular trichomes in several representative plants. We emphasize the regulatory roles of plant hormones, transcription factors, the cell cycle and epigenetic modifications in different stages of trichome development. Finally, we identify the obstacles and key points for future research on plant trichome development, and speculated the development relationship between the salt glands of halophytes and the trichomes of non-halophytes, which provides a reference for future studying the development of plant epidermal cells.

## Introduction

Trichomes are visible on leaves, stems and flower organs of many terrestrial plants (Szymanski et al., 2000; Yang and Ye, 2013; Fei et al., 2020). These hair-like organs derive from a proliferation of epidermal cells, which undergo cell division, differentiation, and growth to produce tissues that extend from the surface of the epidermis. The resulting structures play a variety of roles in plant growth and stress tolerance (reviewed by Balkunde et al., 2010; see also Fei et al., 2020; Mirnezami et al., 2020). For example, trichomes provide a barrier that must be broken before any successful pathogen or herbivore attack; as such, they constitute the first line of plant defense. Trichomes are found in most angiosperms and some gymnosperms and bryophytes (Schuurink and Tissier, 2020; Spirina et al., 2020). Trichome variation is the result of natural selection (Mauricio, 1998; Sletvold et al., 2010; Züst et al., 2012). During evolution, plants have evolved different epidermal structures and their derived structures, such as trichomes and thorn-like leaves, in order to resist various biotic and abiotic stresses (Han et al., 2016). Compared with individuals without trichomes, individuals with trichomes have advantages, particularly in herbivore-rich environments and arid areas (Han et al., 2016; Ying et al., 2018).

Trichomes come in different shapes, sizes and densities. Morphologically, trichomes can be unicellular, bicellular, or multicellular (Andrade et al., 2017; Fei et al., 2020). They may be glandular or non-glandular, and branched or non-branched (Wang et al., 2013b; Shang et al., 2020). Some trichomes are obvious, such as the multicellular trichomes in nettles or tomatoes (*Solanum lycopersicum*), and cause the appearance of a “hairy plant,” while others are small, such as the shield coat of mint species (Turner et al., 2000; Deore et al., 2021).

Unicellular trichomes have a simple structure and usually do not have glands. Non-glandular trichomes are present in most angiosperms, some gymnosperms and bryophytes. However, multicellular trichomes are usually complex. For example, the trichomes of cucumber (*Cucumis sativus*), tobacco (*Nicotiana tabacum*), tomato and snapdragon (*Antirrhinum majus*) are multicellular (Yang and Ye, 2013; Tan et al., 2020; Zhang et al., 2021f). Some multicellular trichomes are secretory and some are not (Koudounas et al., 2015; Xue et al., 2019; Konarska and Łotocka, 2020).

Although such non-glandular hairs do not have secretory or metabolic functions (Yuan et al., 2020), they play an important role in resisting extreme environments, inducing pollination, preventing ultraviolet radiation, resisting drought, adapting to high salinity, absorbing heavy metals and preventing biological invasion and mechanical damage (Riddick and Simmons, 2014; Wang et al., 2020b; Zhang et al., 2021b). For example, cotton (*Gossypium hirsutum*) trichomes (cotton fibers) play an important role in resisting pests such as cotton boll weevil (Ye et al., 2020). Non-glandular hairs can affect herbivorous arthropods and prevent them from climbing over the leaf surface. The hooked tip of non-glandular hairs sometimes hinders their movement, interfering with their feeding or trapping them (Giuliani et al., 2020). After salt treatment, especially moderate and severe salt treatment, *Schizonepeta tenuifolia* increased its density of total trichomes on both sides of leaves to alleviate the effect of salt concentration (Ying et al., 2018). At a low temperature, the protoplast flow of *Saintpaulia ionantha* trichomes stopped immediately, and the aggregation of chloroplasts in cells slowed down. After heating to 20°C, the flow state recovered slowly (Saltveit and Hepler, 2004).

Secretory trichomes, also known as glandular hairs, are usually composed of three parts: the base of the trichome, the gland, and the head of the trichome. Only one cell in the base is tightly connected to the epidermis. The gland is composed of 1–5 short or long cylinder cells, which lack chloroplasts in the cytoplasm. The secretory process of the plant epidermis is closely related to the physiological activities and interactions of various organelles in the cell (Stratmann and Bequette, 2016). Functionally, glandular hairs have the ability to synthesize, store and secrete many specialized metabolites, many of which are of commercial importance in food additives, drugs, flavors, and natural pesticides (reviewed by Feng et al., 2021b). Many are also signal molecules, regulating plant growth and development (Rodziewicz et al., 2019). For example, the chemicals secreted by glandular hairs of crops can repel or even trap insects and mites, resulting in their death due to drying or starvation (Saltveit and Hepler, 2004; Feng et al., 2021b).

Bicellular trichomes are present in plants such as rice and maize (Angeles-Shim et al., 2012; Kong et al., 2021). They are arranged in a cylindrical shape between the stem veins of the upper and lower epidermis of the leaves (Angeles-Shim et al., 2012). Rice trichomes can be subdivided into long hairs, hook hairs, thorn hairs, slender hairs and serrated hairs. Bicellular trichomes also play an important role in plant biotic and abiotic stress responses.

Plant trichome development is influenced by genetic factors and environmental factors such as hormones, water and light (reviewed by Khan et al., 2021). The effects of hormones, water, and light on trichome development have been demonstrated in detail in previous publications (Ning et al., 2016; Li et al., 2021b; Yu et al., 2021b). The trichome pattern formation process has been well studied, mainly because trichomes can easily be observed and used in experiments (Han et al., 2016). Trichomes are an excellent model system for studying cell differentiation, cell cycle regulation, cell polarity, and cell expansion (Stratmann and Bequette, 2016). Therefore, trichomes are often analyzed at the genetic, genomic, and cellular level (Schellmann and Hülskamp, 2005; Xiao et al., 2021). Various mutants have been used to study trichome cell cycle regulation and cell morphogenesis (Aashima et al., 2014; Chen et al., 2021). Plant trichome development generally includes three stages: (1) fate determination and initiation, (2) branching, and (3) elongation and maturation. Many studies have confirmed that different transcription factor families, such as HD-ZIP type proteins (Henriksson et al., 2005; Xie et al., 2021a; Zhang et al., 2021c), C2H2 zinc finger proteins (Gan et al., 2007; Liao et al., 2021), basic helix-loop-helix (bHLH) type proteins (Payne et al., 2000; Liu et al., 2021), and v-myb avian myeloblastosis viral oncogene homolog (MYB) family proteins (Larkin et al., 1994; Khan et al., 2021), all play a key role in plant trichome development. In addition, trichome development is strictly regulated by a variety of plant hormones (Chang et al., 2018; Du et al., 2020; Han et al., 2020). Hormone signaling regulates the formation of trichomes by regulating the expression of downstream genes. Epigenetic modifications also play an important role in plant trichome development (Zhang et al., 2017b; Wu et al., 2020a).

In this paper, we review the molecular regulatory mechanisms of unicellular, bicellular and multicellular trichome development in different plants. We also propose future research directions.

## Molecular Mechanisms of Unicellular Trichome Development

### Trichome Development in *Arabidopsis*

*Arabidopsis* has both unicellular and non-glandular trichomes, which have been systematically studied as models for epidermal cell differentiation. The cell walls of *Arabidopsis* trichomes gradually become thinner from top to bottom. Upon invasion by external organisms or abiotic stress, the highly sensitive base quickly initiates a defense response (Han et al., 2016). In addition, *Arabidopsis* mutants with more trichomes showed less sensitivity to UV compared to wild-type plants, while mutants with fewer trichomes are more sensitive to UV, highlighting that trichomes have an important shielding effect against UV radiation (Riddick and Simmons, 2014; Karabourniotis et al., 2020).

In *Arabidopsis*, trichomes are present in most aerial organs, such as rosette leaves, stems, stem leaves and sepals, but not on hypocotyls and cotyledons (Akhtar et al., 2017). Most studies on the development of trichomes focus on the rosette leaves. Trichomes are single and branched cells covering the whole leaf surface. The trichomes of *Arabidopsis* are developed from a single protoepidermal cell at the leaf base. They are usually separated by three to four epidermis cells. As leaves grow, new trichomes form at the base of leaves, while the existing trichomes separate due to the division of intermediate epidermal cells (Banyar et al., 2015). The morphological photographs of *Arabidopsis* trichomes were shown in Supplementary Figure 1A (Breuer et al., 2009). The occurrence of trichomes is the result of external signals and endogenous transcriptional regulation (Hülskamp et al., 1994; Pesch and Hülskamp, 2009; Maes et al., 2011). As early as the 1990s, more than 70 *Arabidopsis* trichome mutants have been isolated; they can be divided into the following six types: with trichomes, without trichome, trichome ribbons, trichome reduction, trichome distortion, and vitreous trichomes (Hülskamp et al., 1994). Over the past few decades, researchers have focused on the molecular mechanisms of a series of trichome mutants such as *gl1*, *gl2*, and *ttg1* (Oppenheimer et al., 1991; Rerie et al., 1994; Walker et al., 1999). With the discovery of novel genes regulating trichome development, new mutants at various stages of trichome development, such as *gis*, *kak*, and *etc1* were complemented in the mutant library (Kirik et al., 2004; Yan et al., 2012; Bensussan et al., 2015). Many important genes that regulate the development of trichomes have been discovered, most of which are transcription factors (reviewed by Schwab et al., 2000; Han et al., 2021).

Based on the study of the hair development process in *Arabidopsis*, we divided the development of *Arabidopsis* trichomes into three stages: fate determination and initiation, trichome branching, and trichome elongation and maturation (reviewed by Payne et al., 1999; see also Hülskamp, 2004; Schellmann and Hülskamp, 2005). Morphogenesis can be divided into six stages, beginning which the trichome cell expanding rapidly relative to the surrounding normal epidermal cells and ending with a circle of trichome-supporting cells surrounding the trichome (Szymanski and Marks, 1998).

In the fate determination and initiation stage, positive regulators, such as R2R3-MYB transcription factors, WD40 (WD40 repeat) proteins, bHLH transcription factors and C2H2 zinc finger proteins, and negative regulators, such as R3-MYB transcription factors, play a key role in this process (Zang et al., 2015; Han et al., 2021).

C2H2-type zinc finger proteins play a key role in upstream trichome fate determination and initiation in *Arabidopsis*. *GLABROUS INFLORESCENCE STEMS* (*GIS*), *GIS2*, *GIS3*, *ZINC FINGER PROTEIN 1* (*ZFP1*), *ZFP5*, *ZFP6*, and *ZFP8* all encode C2H2 zinc finger proteins that play important roles in trichome development (Gan et al., 2007; Zhou et al., 2011, 2012, 2013; Sun et al., 2015a; Liu et al., 2017; Zhang et al., 2020; Han et al., 2021). *GIS* functions upstream of the trichome initiation complex GLABRA1 (GL1)-GLABRA3 (GL3)-TRANSPARENT TESTA GLABRA 1 (TTG1) (GL1-GL3-TTG1) and downstream of the gibberellin (GA) signal repressor *SPINDLY* (*SPY*). It responds to GA and promotes inflorescence trichome initiation. This pathway is negatively regulated by *SPY* and the DELLA repressor *GIBBERELLIC ACID INSENSITIVE* (*GAI*) (Guan et al., 2007). *GIS3* receives GA and cytokinin (CTK) signal transduction and directly targets the downstream genes *GIS* and *GIS2* to regulate the production of trichomes (Sun et al., 2015a). *ZFP6* responds to GA and CTK signaling and plays a role upstream of *ZFP5* (Zhou et al., 2013). *ZFP5* acts upstream of the *GIS* gene family and key trichome initiation regulators, participates in GA signal transduction and controls the development of trichomes. *ZFP8* is also a direct target gene of *ZFP5* (Zhou et al., 2011, 2012). In addition, the function of the protein encoded by *ZFP5* in controlling trichome initiation is equivalent to that of GIS and GIS2. They all play critical roles upstream of the trichome initiation complex GL1-GL3-TTG1. CTK induces the expression of *ZFP1*, thereby increasing the expression of *GL3* and finally promoting trichome initiation in *Arabidopsis* (Sun et al., 2015a; Xie et al., 2019; Zhang et al., 2020). TRICHOME-RELATED PROTEIN (TRP) is also a recently identified C2H2 zinc finger protein, which negatively regulates trichome initiation-related transcription factors through GA signaling. TRP can interact with ZFP5, preventing ZFP5 from binding to the *ZFP8* promoter and inhibiting the occurrence of superficial trichomes (Kim et al., 2018).

Downstream of C2H2 zinc finger proteins, *GL1* was the first factor found by researchers to control the development of plant trichomes (Oppenheimer et al., 1991). It encodes an MYB-like protein, and knockout of this protein will produce hairless leaves (Ram et al., 2015). *GL3* encodes a bHLH transcription factor and has redundancy with *ENHANCER GLABRA3* (*EGL3*) (Payne et al., 2000; Zhang et al., 2003; Hung et al., 2020). A double mutation of *GL3* and *EGL3* leads to a trichome defect. *TRANSPARENT TESTA GLABRA* (*TTG*) has been studied since the 1990s (Larkin et al., 1994, 1999). *TTG1* encodes a protein with 4–5 repeating WD-40 motifs, interacts with *GL3*, and ultimately promotes trichome differentiation (Walker et al., 1999; Champagne and Boutry, 2017). The semi deletion alleles of *GL1* and *TTG1* produce aborted trichomes, indicating that *GL1* and *TTG1* function as complexes and dual regulators in trichome development (Jose et al., 2018; Hung et al., 2020). *SUPER SENSITIVE TO ABA AND DROUGHT2* (*SAD2*) encodes an importin β-domain protein that has the same function as GL1, GL2 and GL3 in regulating trichome development (Gao et al., 2008; Du et al., 2018). *SAD2* can mediate the function of *GL3* and regulate the expression of *GL1, TTG1* and *GL2* (Gao et al., 2008). *TRIPTYCHON* (*TRY*) and *CAPRICE* (*CPC*) negatively regulate the development of trichomes; they mainly encode R3-MYB transcription factors (Wada et al., 1997; Szymanski and Marks, 1998; Champagne and Boutry, 2017). They can competitively bind to the N-terminal of *GL3* and *EGL3*, damage the function of *GL3-* and *EGL3*-related complexes, and interfere with the differentiation and development of trichomes (Feng et al., 2017). The R3-MYB transcription factors ENHANCER OF TRY AND CPC 1 (ETC1) inhibit the fate of *Arabidopsis* aboveground trichomes and non-hairy root epidermal cells (Victor et al., 2004). *etc1* mutants have no distinct phenotype but enhance the roles of *cpc* and *try* in trichome development. *etc1 try cpc* triple mutants produce more trichomes on the upper epidermis of leaves and hypocotyl than the wild type, *etc1*, *try*, *cpc*, *etc1 try* double mutant, *etc1 cpc* double mutant and *try cpc* double mutant (Victor et al., 2004). *SQUAMOSA PROMOTER BINDING PROTEIN LIKE* (*SPL*) gene is also a negative regulator of trichome development. SPL temporally controls trichome distribution during flowering. Increasing *SPL* transcription levels were associated with progressive loss of stem hair cells. SPL9 directly activates its expression by binding to *TCL1* and *TRY* promoters, which is independent of GL1; GIS-dependent pathways do not affect SPL9 regulation of TCL1 and TRY (Yu et al., 2010). SPL is targeted by microRNA 156 (miR156); overexpression of miR156 results in ectopic trichomes on the stem and flower organs of plants, while an increased SPL expression level in plants results in less trichomes than that in the wild type (Yu et al., 2010; Wei et al., 2012). TTG2, the first WRKY transcription factor associated with the mutant phenotype, regulates the development of trichomes, seed coats and root hairs (Johnson et al., 2002). Analysis of trichome and root hair mutants showed that the expression of *TTG2* is regulated by MYB and bHLH genes. *TTG2* mutations lead to phenotypic defects in trichome development (reviewed by Ishida et al., 2007). Most leaf surface hairs in *ttg2* mutants are unbranched (> 95%), and the number of trichomes formed per leaf is approximately halved, and even glassy and twisted trichomes appear (Johnson et al., 2002). *GL2*, a homeobox family gene that encodes the HD-ZIP IV transcription factor, plays an essential role in and is expressed throughout trichome development (Rerie et al., 1994; Bryant et al., 2016). The upstream pathway of *GL2* regulating the differentiation and development of plant epidermal cells is relatively clear. The R2R3-MYB transcription factor GL1/MYB23, WD40 protein TTG1 and bHLH transcription factor GL3/EGL3 together form a trimeric complex activator, which directly acts on the downstream GL2/TTG2 to regulate trichome development. However, the regulatory pathway downstream of *GL2* is not clear. GL2, as a transcription factor, may affect cell differentiation and development by regulating the expression of downstream target genes. Studies have shown that knockdown of the bHLH-type transcription factor AtMYC1 reduces the density of *Arabidopsis* trichomes (Symonds et al., 2011). Further studies showed that *MYC1* could regulate the intracellular localization of *GL1* and *TRY*, suggesting that *MYC1* may inhibit the activity of *CPC* and *TRY*, thereby regulating the number of trichomes (Pesch et al., 2013).

In the branching stage of trichomes, branching is regulated by microtubules, and the arrangement direction of microtubules controls the growth direction and branching ability of trichomes (Tominaga-Wada et al., 2012). The DNA in trichome nuclei needs four replications to form a branching structure. *SIAMESE* (*SIM*), *CONSTITUTIVE EXPRESSION OF PR GENES 5* (*CPR5*) and *RETINOBLASTOMA-RELATED* (*RBR*) negatively regulate trichome branching by controlling nuclear replication (Kirik et al., 2001; Wang et al., 2021c). SIM is a cell cycle mitotic inhibitor of endonuclear replication (Walker et al., 2000). The *sim* mutant has fewer branched trichomes than the wild type because the transition of trichome cells from mitosis to intranuclear replication is inhibited (reviewed by Walker et al., 2000; see also Churchman et al., 2006; Grebe, 2012). *SIM* is likely a direct target for *GL3* to control intracellular replication. In a chromatin immunoprecipitation (ChIP) experiment, *GL1* and *GL3* bound to the promoter region of *SIM* (Walker et al., 2000). *CELL CYCLE SWITCH PROTEIN 52 A1* (*CCS52A1*) and *SIM* cooperate to inhibit the accumulation of mitotic cyclin to establish the inner loop of trichomes (Remmy et al., 2010). *MYB5* and *MYB23* are members of the R2R3 MYB family that regulate trichome extension and branching. The *myb5* mutant showed little change in trichome morphology, whereas the *myb23* mutant produced more small trichomes and two branched trichomes. The *myb5 myb23* double mutant had shorter trichomes and more double-branched trichomes on rosette leaves than the single mutant (Li et al., 2009). In addition, the R2R3-MYB transcription factor MIXTA gene *AtMYB106* negatively regulates the *SIM* gene (Jakoby et al., 2008; Tian and Zhang, 2021). *CPR5* encodes a protein of unknown function that might be a membrane-bound protein. Compared with wild type, *cpr5* mutants have smaller trichomes, less branching, and lower nuclear DNA content. In *cpr5* mutant trichomes, the endonuclear replication cycle stops after two rounds instead of four, and the trichome cells are less branched than normal. The *cpr5* mutant also has altered cell walls and reduced cellulose content in leaves and trichomes (El Refy et al., 2003; Brininstool et al., 2008). DA3-encoded *UBIQUITIN-SPECIFIC PROTEASE 14* (*UBP14*) acts upstream of *CYCLIN-DEPENDENT KINASE B1;1* (*CDKB1;1*), affecting endonuclear replication and cell growth in *Arabidopsis*. The *da3-1* inhibitor *SUPPRESSOR OF DA3-1 6*; *SUD6*, which encodes CYCLIN-DEPENDENT KINASE G2 (CDKG2), promotes nuclear replication and cell growth and ultimately increases the number of trichome branches (Jiang et al., 2022). Studies have shown that *TRY* and *ETC1* genes cooperate to regulate trichome branches. The trichomes of *try etc1* double mutants have more branches than wild type and *try* single mutants. *ETC1* can enhance the function of *TRY* to regulate the number of trichome branches (Tominaga-Wada and Nukumizu, 2012). In *cpl3* mutants, the level of inscribed replication in the epidermis increases, resulting in a reduction of trichome branches (Tominaga-Wada and Nukumizu, 2012). Furthermore, *GIS* plays an inhibitory role in trichome branching, acting downstream of the key regulators *STICHEL* (*STI*) and *SIM* (Yan et al., 2014). Class I TCP transcription factors TCP14 and TCP15 regulate hyperdivided trichomes and increase stratum corneum permeability in *Arabidopsis*. *TCP14* inhibits trichome branching in *Arabidopsis* leaves and inflorescence stems by direct transcriptional activation of *GIS* (Vadde et al., 2018). The protein encoded by *AtTCP15* binds directly to the promoter regions of *CYCLIN A 2;3* (*CYCA2;3*) and *RBR* genes and plays a key role in endonuclear replication (Li et al., 2012b). *ANGUSTIFOLIA* (*AN*) enriches high concentrations of microtubules at the top of trichome cells (Kim et al., 2002; Hülskamp, 2004; Hashida et al., 2020); *ZWICHEL* (*ZWI*) is involved in the initiation of trichome branching (Reddy et al., 2004; Chen et al., 2016b); *STI* and *TUBULIN FOLDING COFACTOR A/C* (*TFCA/C*) are involved in the formation of trichome branching (Victor et al., 2002); and *FASS/TONNEAU2* (*TON2*) and *SPIKE* are involved in trichome branching by regulating microtubule tissue (Traas et al., 1995; Qiu et al., 2002; Deeks and Hussey, 2003; Ilgenfritz et al., 2003). *INHIBITOR/INTERACTOR OF CDK 1* (*ICK1*)/*KIP RELATED PROTEIN 1* (*KRP1*) positively regulates trichome branching. Overexpression of *ICK1*/*KRP1* in *Arabidopsis* reduces intranuclear replication and cell size and induces cell death (Ilgenfritz et al., 2003; Schnittger et al., 2003; Wang et al., 2021c). *ROOT HAIRLESS 2* (*RHL2*) and *HYPOCOTYL6* (*HYP6*) encode subunits of DNA topoisomerase VI, indicating the existence of DNA replication pathways specific to internally replicating cells (Sugimoto-Shirasu et al., 2002). In *rhl2*, *hyp6*, and spindly (*spy*) mutants, trichomes are smaller, defective, branched, and have lower nuclear DNA content than in the wild type (Jacobsen and Olszewski, 1993; Sugimoto-Shirasu et al., 2002). In *kaktus* (*kak*) mutants, trichome branching increases (El Refy et al., 2003; Schellmann and Hülskamp, 2005).

During the elongation and maturation stage, trichome lengths increase to several times their previous length and expand in a polarized manner. The actin cytoskeleton determines the direction of trichomes cell expansion. Genes such as *BRICK1* (*BRK1*), *DISTORTED* (*DIS*) and *ROP* are involved in trichome cell actin cytoskeleton expansion (Hülskamp et al., 1994; Yang, 2002). The mutation of *Arabidopsis BRK1* gene leads to the morphological defects in trichome cells and related changes in the F-actin cytoskeleton. *BRK1* is necessary for SCAR protein accumulation *in vivo*, which may explain the important role of *BRK1* in *ARP2*/*3* complex function (Frank and Smith, 2002). *ARP2*/*3* is a large complex that promotes actin formation (Dipanwita et al., 2004; Papalazarou and Machesky, 2021). The following genes, known as DIS genes, encode components of the ARP2/3 complex: *WRM*, *CRK*, *DIS2*, *DIS1*, *PIR*, *KLK*, *GRL* and *ITB1*. Mutants of these genes have altered trichime elongation and growth direction; they have a phenotype of epidermal torsion, with some areas of cells bulging and displaying dysplasia (Hülskamp et al., 1994; Yang, 2002). A small GTPase called ROP controls downstream genes to regulate actin configuration in trichome cells. ROP binds to the ARP2/3 complex to release PIR121, NAP125, and ABI2, resulting in the activation of HSPC300 and SCAR/WAVE (Dipanwita et al., 2004).

Environmental and endogenous signals closely regulate trichome development. Several plant hormones, including those mentioned above (GA and CTK) are involved in trichome differentiation in *Arabidopsis*. Application of jasmonic acid (JA) leads to significantly more leaf trichomes (Qi et al., 2011). Salicylic acid (SA) can reduce the number of trichomes (Traw and Bergelson, 2003). CTK and GA stimulate the development of trichomes on inflorescence stems (Fan et al., 2020; Papalazarou and Machesky, 2021). In addition, plant hormones can also play an important signaling role in trichome development by mediating downstream genes (reviewed by Markus, 2012). GAI and *SPY* are a GA biosynthesis factor and a GA signal inhibitor, respectively. Mutations in these have a significant impact on epidermal hair development. There is a positive relationship between GA level and trichome development. GAs may rely on *GL1* and *TTG* to promote trichome development (An et al., 2012). *TEM1* and *TEM2* encode members of ABI3 and VP1 transcription factor families. *TEM1* and *TEM2* play a negative regulatory role in trichome development, which depends on the GA pathway. TEMs not only regulate GA content but also the transportation and distribution of GA in mesophyll, which mediates trichome development. This indicates that subepidermal cells play a role in trichome initiation (Fiehn et al., 2000). A ChIP experiment showed that TEMs directly bind to the promoters of *GL1*, *GL2*, *GIS2*, and *ZFP8*, which may regulate and transcriptionally inhibit some transcription factors of coding genes in the initial stage of trichome development. As an important inhibitor of the JA signaling pathway, *JASMONATE ZIM-DOMAIN PROTEIN 1* (*JAZ1*) can degrade under the action of JA, release MYB-bHLH-WD40 activity and promote trichome development. *NUCLEOREDOXIN 2* (*NRX2*) promotes JA-mediated trichome formation in *Arabidopsis*; trichome formation is significantly reduced in the *nrx2* mutant compared to in the wild type. *JMJ29* is a histone demethylase-containing JMJC domain belonging to the JHDM2/KDM3 group. It participates in epidermal hair development by directly regulating *GL3* expression (Fuyu et al., 2020). The cell cycle regulator *CPR5* is involved in the SA signal pathway. Trichome length and number of branches are lower in *cpr5* mutants than in the wild type (Kirik et al., 2001). These mutants also contain high SA content, suggesting that *CPR5* may also be involved in SA biosynthesis (Peng et al., 2020).

Epigenetic modification is an important regulation mode, it is involved in a variety of protein post-translational modifications, such as ubiquitination, acetylation, methylation, glycosylation, and DNA methylation (Scoville et al., 2011). In addition, non-coding RNA, as an epigenetic regulator, also plays an important role in plant growth. Recent studies suggest that the multifunctional histone acetyltransferase AtGCN5 may have a positive effect on trichome branching by regulating *TRY* (Hülskamp et al., 1994). *GCN5* also participates in the regulation of epidermal initiation through histone acetylation acting on the promoters of *GL1*, *GL2*, *GL3*, and *CPC*. Free degradation and mutant analysis of plants showed that *UBIQUITIN PROTEIN LIGASE 3* (*UPL3*) promotes *GL3* and *EGL3* degradation, which in turn inhibits epidermal branching. As a histone chaperone, CAF-1 participates in trichome development through a pathway independent of internal duplication (reviewed by Hussey et al., 2006). In eukaryotes, the modification of mRNA is related to cell development and differentiation, in which *N6*-methyladenosine (m6A) is the most common genomic marker. Similar to DNA methylation, known regulatory genes can express m6A, and factors of different functions can also write, clear and read m6A (Wu et al., 2020a). ECT2 is an interpretation protein of m6A. It binds and stabilizes key transcripts related to trichomes, such as *TTG1*, affecting trichome development. This provides the groundwork for future research on post transcriptional modification as a key factor in trichome development (Wei et al., 2018b; Arribas-Hernández et al., 2021).

**FIGURE 1:**
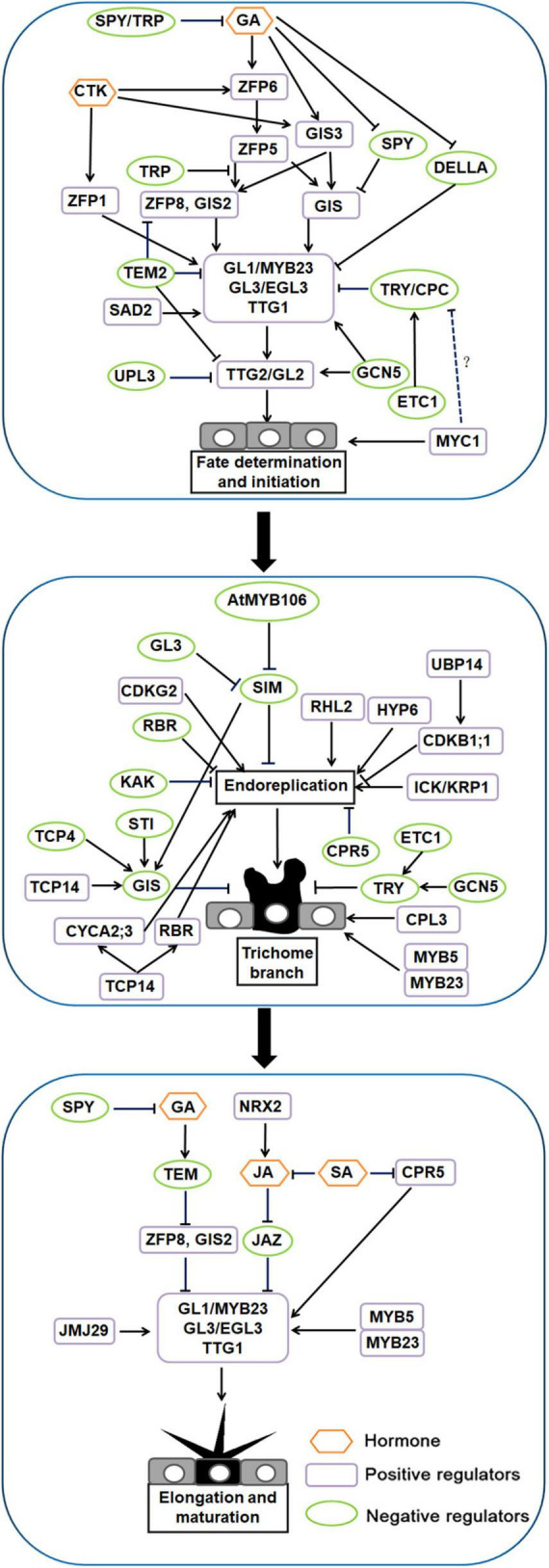
Regulatory network model for trichome development in *Arabidopsis*.

A model of trichome fate determination and initiation, branching, and elongation and maturation in *Arabidopsis* is shown in Figure 1.

### Trichome (Fiber) Development in Cotton

Trichomes on cotton seed coats are usually called cotton fiber. Morphological photographs of cotton fibers were shown in Supplementary Figure 1B (Guan et al., 2014b). Cotton fiber is the main harvest of cotton and an important raw material for the textile industry. Cotton fiber development includes the following four main stages: initiation, elongation, secondary cell wall deposition and maturation (Hao et al., 2018).

Regulation of cotton fiber initiation [2–5 days after anthesis (DPA)] is similar to that of *Arabidopsis* trichomes, with several similar transcription factors and hormone signals.

Several cotton genes encoding MYB-type transcription factors that are homologous with *Arabidopsis* genes have been cloned and identified. *GhMYB2* has a similar sequence to *Arabidopsis GL1* and controls cotton fiber cell fate determination (Wang et al., 2004; Yong-Mei and Yu-Xian, 2011; Andreas et al., 2014; Zhao et al., 2020). The *GhMYB2* gene promotes cotton fiber development and has functional homology with *Arabidopsis GL1* in terms of trichome formation. Allotetraploid cotton containing *GhMYB2A* and *GhMYB2D* homologs is currently the most widely grown cotton variety. Cotton accumulates less *GhMYB2A* mRNA than *GhMYB2D* during the fiber initiation stage. *GhMYB2D* mRNA is targeted by miR828 and miR858, resulting in trans-siRNAs (ta-siRNAs) in the TRANS-ACTING SIRNA GENE 4 (TAS4) family. Four families of genes encoding ta-siRNA were found in *Arabidopsis*, namely TASl, TAS2, TAS3, and TAS4. TAS4 was discovered when a special algorithm clustered 21-base fragments within the genome. After the primary transcript of TAS4 was recognized by miR828, it was cleaved to generate the corresponding ta-siRNA. miR828 directs the cleavage of RNA derived from TAS4 and initiates the production of phasic small interfering RNA (siRNA) dependent on RNA-DEPENDENT RNA POLYMERASE 6 (RDR6) (Bonar et al., 2018). Overexpression of *GhMYB2A* but not *GhMYB2D* complements *gl1* phenotype. Mutation of the miR828 binding site or replacement of its downstream sequence can eliminate the production of ta-siRNA and restore trichome development in the *gl1* mutant. In addition, blocking the biogenesis gene *DICER-LIKE 4* (*DCL4*) or *RDR6* of ta-siRNAs in *gl1 GhMYB2D* overexpressing body can restore trichome development (Voinnet, 2009). The functional difference between *GhMYB2A* and *GhMYB2D* homologous genes is caused by ta-siRNA mediated by miR828, which indicates the unique role of microRNA in the functional difference of target homologous genes. It also indicates that the evolution and selection of morphological characteristics of target homologous genes are vital. Small RNAs, including miRNAs and ta-siRNAs, are involved in the regulation of gene expression and development in plants and animals. In *Arabidopsis*, ta-siRNA biogenesis is usually triggered by miRNA cleavage at the TAS site (Allen et al., 2005). RNA-induced silencing complexes associated with the ARGONAUTE proteins AGO1 and AGO7 are assembled to target sites to cleave transcripts (Montgomery et al., 2008). Cleaved mRNA fragments serve as templates for RNA-dependent RNA polymerase 6 (RDR6), producing double-stranded RNAs (Peragine et al., 2004; Vazquez et al., 2004), which can be recognized by DCL4 and processed into 21-nt siRNAs or ta-siRNAs20. ta-siRNA regulates the development of trichomes in *Arabidopsis* leaves and the development of cotton fibers. *GhMYB3* is a functional homolog of *GL1* and *GhMYB2*. *GhMYB3* interacts with *Arabidopsis* GL3 protein to regulate *Arabidopsis* trichome development. Ectopic expression of *GhMYB3* rescues the hairless phenotype of the *Arabidopsis gl1* mutant, produces more ectopic trichomes in the stems and flower organs of the inflorescence, and has an orthologous function in the development of plant trichomes (Shangguan et al., 2021). *GhMYB25* and *GhMYB25*-like also play a positive regulatory role in fiber initiation. *GhMYB25*-like plays a role upstream of *GhMYB25* and *GhMYB109* (Wang et al., 2021a). *GhMYB109* was found to be involved in the initiation and differentiation of fiber by RNAi experiments (Suo et al., 2003). *GhMYB109* is structurally similar to *GL1* and *WER*; which control *Arabidopsis* trichome initiation and have strong similarity to the R2R3 domain of *GL1*. *GhMYB109* plays a positive regulatory role in the initiation and elongation of cotton fiber and affects the expression of downstream genes including *GhACO1*, *GhACO2*, *GhTUB1*, and *GhACT1* (Suo et al., 2003). The gene encoding 1-aminocyclopropane-carboxylate oxidase (ACO), the last rate-limiting enzyme in ethylene (ETH) biosynthesis, plays a role in fiber development and is positively correlated with fiber elongation, maintaining ETH during fiber development (Wang et al., 2011; Cong et al., 2017; Li et al., 2021c). The MYB transcription factor GhCPC is homologous to *Arabidopsis CPC* and negatively regulates cotton fiber elongation. Transgenic evidence suggests that overexpression of *GhCPC* results in a delay in fiber initiation and a reduction in fiber length (Prakash et al., 2020). A yeast two-hybrid analysis showed that *GhCPC* can interact with the bHLH-type protein GhMYC1, and *GhMYC1* can also interact with *GhTTG1* and *GhTTG4*. *GhCPC* negatively regulates early fiber initiation and elongation through the CPC-MYC1-TTG1/4 complex (Liu et al., 2015; Zhang et al., 2021d). In the 35S*:GhCPC* transgenic line, the transcription levels of downstream genes *GhHOX3* and *GhRDL1* decreased significantly compared to in the wild type (Deng et al., 2012a). The promoter of the cotton dehydration-responsive gene *RDL1* contains homeodomain-binding and MYB-binding motifs, which can specifically express trichomes of *Arabidopsis* (Wang et al., 2004; Chen et al., 2015).

HD-ZIP-type transcription factors also play an important regulatory role in cotton fiber initiation. *PROTODERMAL FACTOR1* (*GbPDF1*) encodes the homologous framework HD-ZIP protein, which plays a role in fiber initiation and early elongation by interacting with PPIP1, PPIP2, and PPIP3. Knockout of *GbPDF1* can result in delayed fiber initiation, fiber shortening, and decreased lint percentage, indicating that *GbPDF1* plays an important role in fiber development (Jiang et al., 2012; Wang et al., 2019d). *GbPDF1* is also involved in the homeostatic regulation of H_2_O_2_ during fiber development. Inhibiting the expression of *GbPDF1* results in a substantial accumulation of H_2_O_2_ and delays the development of cotton fibers (Deng et al., 2012a). The HD-ZIP IV family transcription factor GOSSYPIUM BARBADENSE MERISTEM LAYER 1 (GbML1) interacts with GhMYB25 and specifically binds to the L1-box of the dehydration-inducing protein *GbRDL1* promoter (Zhang et al., 2010). GaHOX1 is a class IV HD-ZIP transcription factor. When expressed under the control of the *GL2* promoter, *GaHOX1* rescues the trichome development of *gl2-2 Arabidopsis* hairless mutants. This indicates that *GaHOX1* is a functional homolog of *GL2* in the development of plant trichomes (Humphries et al., 2005; Guan et al., 2008; Zhang et al., 2010). The HD-Zip protein GhHD1 positively regulates cotton fiber formation by regulating reactive oxygen species (ROS) and ETH accumulation (Walford et al., 2012).

bHLH-type transcription factors are also involved in cotton fiber initiation. GhMYC1 is a bHLH-type transcription factor homologous to *GL3*; it can bind to the E-box sequence of the *GhHOX1* promoter, indicating that *GhHOX1* may be located downstream of *GhMYC1* in fiber development regulation (Hu et al., 2018). In addition, the ectopic expression of the bHLH transcription factor GhDEL65 increases trichome density; it likely regulates cotton fiber development by interacting with *GhMYB2* and *GhMYB3* (Shangguan et al., 2016; Sun et al., 2020).

GhWRKY16 is a WRKY transcription factor; it can directly bind to the promoters of *GhHOX3*, *GhMYB109*, *GhCesA6D-D11*, and *GhMYB25* to induce the expression of these genes, thereby promoting fiber initiation and elongation (Wang et al., 2021b).

The expression of *VACUOLAR INVERTASE* (*VIN*) is necessary for cotton fiber initiation, and RNAi-mediated inhibition of *GhVIN1* leads to a significant decrease in VIN activity, thereby forming a fiber-free seed phenotype in a dose-dependent manner (Wang et al., 2014). *GhVIN1*-mediated hexose signaling acts upstream of *GhMYB25-like*, *GhMYB25*, and *GhMYB109* transcription factors (Prakash et al., 2020).

Plant hormone signaling pathways also play an important role in the regulation of cotton fiber initiation. Auxin (AUX) biosynthesis and transport plays a major role in the accumulation of AUX in the ovule and fiber. The AUX efflux mediated by the IAA efflux transporter (PIN) in the ovule participates in the accumulation of AUX and the development of cotton fiber (Zhang et al., 2017b; Ma et al., 2019). Excessive *GhPIN3* transcripts in the epidermis of the ovule promote the accumulation of fiber-specific AUX, thereby promoting fiber initiation, which indicates that all AUX signal transduction pathway components divide labor and play an important role in fiber development (Li et al., 2007; Zhang et al., 2017a). The AUX response factor *ARF* is expressed in multiple cotton tissues. *GhARF2b*, the *Arabidopsis AtARF2* homolog, is preferentially expressed in developing ovules and fibers (Liu et al., 2020a; He et al., 2021). Overexpression of *GhARF2b* inhibits cotton fiber cell elongation but promotes fiber initiation. However, RNAi lines of *GhARF2b* resulted in fiber reduction but lengthening compared with that in the wild type. *GhARF2b* directly interacts with *GhHOX3* and inhibits the transcriptional activity of *GhHOX3* on target genes (Zhang et al., 2021d). *GhARF2-1* and *GhARF18-1* are only expressed in trichomes, and overexpression of these two genes in *Arabidopsis* enhances trichome initiation (Xiao et al., 2018). Similar to in *Arabidopsis*, GhJAZ2 protein is a negative regulatory protein in the JA pathway. *GhJAZ2* interacts with *GhMYB25-like*, *GhGL1*, *GhMYC2*, *GhWD40*, and *GhJI1* as a mediator of the JA signaling pathway and negatively regulates fiber development in cotton (Guan et al., 2014a). During cotton fiber initiation, JA regulates the downstream gene *GhMYB25-lik*e through *JAZ2*, while *GhCPC* prevents the expression of *TTG1*/*MYC1*, promotes the expression of *GhHOX1*, and initiates the formation of cotton fiber (Zhou et al., 2015). Exogenous ETH can promote the accumulation of H_2_O_2_ and play a positive regulatory function in fiber development, which indicates that ETH has a synergistic effect with the ROS pathway in fiber (Kim et al., 2017). *PAGODA1* (*PAG1*) encodes CYP734A1, which degrades brassinosteroid (BR) through C26 hydroxylation and negatively regulates fiber development. As an acidic protein, Gh14-3-3 not only interacts with *GhBZR1*, but also regulates BR signals, thereby promoting fiber initiation and elongation (Li et al., 2013).

Transcription factors and hormone signaling pathways are also involved in the cotton trichome elongation stage (3–20 DPA).

The MYB transcription factor GhMYB212 is an important factor that regulates the transport of sucrose from the ovule to the fiber. *GhMYB212* also controls expression of the sucrose transporter gene *GhSWEET12*, mediates the transport of sucrose and glucose, and mediates fiber development (Kai et al., 2017). *GhMYB212* promotes the expression of *GhSWEET12*, and then *GhPDF1* promotes the accumulation of ROS, which regulates cotton fiber elongation. During the transition from the initiation to the elongation stage, ETH and ROS mutually mediate cotton fiber initiation and elongation (Sun et al., 2019).

The bHLH transcription factor gene *GhHOX3* is located in the 12th homologous chromosome of allotetraploid cotton varieties and is related to the quantitative trait locus (QTL) of fiber length (Shan et al., 2014). *GhHOX3* can bind to the promoters of the cotton cell wall relaxin genes *GhRDL1* and *GhEXPA1* and activate their expression to promote cotton fiber elongation. The plant hormone GA can modulate the activity of *GhHOX3* (Shan et al., 2014; Shangguan et al., 2021). The GhHOX3–GhHD1 interaction increases the transcriptional activity of *GhHOX3* and its role in fiber elongation. The cotton bHLH transcription factor GhFP1 activates BR biosynthesis and signal transduction to positively regulate fiber elongation (Liu et al., 2020b). GhbHLH13 is a bHLH transcription factor that is upregulated during fiber elongation. *GhPEL76* causes shortening of cotton fibers after virus-induced gene silencing (VIGS). Yeast one-hybrid and transient dual-luciferase assays showed that *GhbHLH13* can activate *GhPEL76* and regulate cotton fiber elongation by binding to the G-box in the promoter region of *GhPEL76* (Sun et al., 2020).

Phospholipids and structural proteins are also involved in cotton fiber elongation. Phosphatidylinositol (PtdIns) is an important structural phospholipid, and exogenous PtdIns application can promote cotton fiber elongation (Long et al., 2018). In transgenic cotton plants, the expression of the cotton PtdIns synthase gene *GhPIS* is controlled by the fiber-specific promoter element, resulting in the specific up-regulation of *GhPIS* during cotton fiber elongation (Long et al., 2018). ARABINOGALACTAN PROTEINs (AGPs) are extracellular proteoglycans that play an important role in intercellular communication during cotton fiber elongation and secondary cell wall formation. Four AGP genes (*GhAGP2*, *GhAGP3*, *GhAGP4*, and *GhFLA1*) have been cloned from cotton fiber (Liu et al., 2008). *GhAGP4* RNAi lines have markedly lower expression levels of *GhAGP4* and partially down-regulated expression of *GhAGP2*, *GhAGP3*, and *GhFLA1* compared to that in the wild type. As a result, fiber elongation is inhibited, reducing fiber quality (Li et al., 2010b). Transcription profile analysis showed that nine beta-tubulin (TUB) genes were highly expressed in elongated fiber cells compared with ovules without villi mutants (Li et al., 2002; He et al., 2008). Northern blot analysis showed that the *GhTUB1* gene is specifically expressed in cotton fiber cells (Li et al., 2003).

Ethylene also plays a vital and active role in cotton fiber elongation. Several genes related to ETH biosynthesis are positively related to cotton fiber development, such as *MAT* (*GA_Ed0052D12f*), which is a homolog of *GhMAT4* (Yu et al., 2021a). Overexpression of the *Arabidopsis* gene *ANKYRIN REPEAT PROTEIN 2A* (*AKR2A*) promotes cotton fiber elongation by increasing ETH biosynthesis and synergistic action with AUX accumulation (Hu et al., 2020). The plant hormones ABA and CTK inhibit fiber growth, and ETH may negatively regulate their abundance to promote fiber development (Yu et al., 2021a). *GhDET2* encodes a rate-limiting enzyme 5α-reductase for BR biosynthesis, which promotes the density and length of cotton fibers (Luo et al., 2007). Gh14-3-3 interacts with GhBZR1 to regulate BR signaling and promote cotton fiber elongation (Zhou et al., 2015). Cotton *GhPAG1* encodes a homolog of *Arabidopsis CPY7341*. The *ghpag1* mutant exhibits a typical phenotype lacking BR. *GhPAG1* potentially regulates cotton fiber by participating in the BR pathway. *GhPAG1* is highly expressed in 15 DPA fibers and regulates fiber elongation by controlling the level of endogenous biologically active BRs (Yang et al., 2014b; Li et al., 2021b). Cotton DELLA protein GhSLR1 interferes with the stability of the GhHOX3-GhHD1 complex, inhibiting the transcription of downstream target genes and subsequent fiber elongation (Xia et al., 2018). GhMADS11 is a MADS transcription factor that can accumulate in cotton fibers and promote cell elongation (reviewed by Wang et al., 2019d). Heterologous overexpression of *GhMAD14* can reduce GA content and shorten the length of *Arabidopsis* hypocotyls compared with that in the wild type (Zhou et al., 2014).

In the fibrous secondary cell wall deposition stage (16–40 DPA), a set of enzymes synthesize secondary cell walls (SCWs). The NAC-MYB-CESAs module defines the central pathway of SCW cellulose biosynthesis.

NAM, ATAF and CUC (NAC) transcription factors act as the main switch in the NAC-MYB-cellulose synthases (CESAs) pathway (Taylor-Teeples et al., 2015; Cao et al., 2020). In *Gossypium hirsutum*, GhFSN1 is a NAC transcription factor; it regulates SCW biosynthesis in cotton fibers. *GhFSN1* positively regulates the formation of cotton fiber SCWs by regulating the expression of its downstream genes *GhIRX12*, *GhMYB1*, *GhGUT1*, and *GhDUF231L1*. Overexpression of *GhFSN1* by the 35S promoter increases cotton fiber SCW thickness and slightly reduces fiber length (Jie et al., 2018; Zhang et al., 2018).

MYB-type transcription factors play an important role in the deposition of fibrous SCWs. Two MYBs, *GhMYB46_D13* and *GhMYB46_D9*, are highly expressed in 20 DPA fibers. Both *GhMYB46_D13* and *GhMYB46_D9* can bind the promoter of the cotton fiber SCW cellulose synthase gene, enhancing its expression and promoting fiber SCW thickening (Huang et al., 2019a). In addition, two cotton R2R3 MYB transcription factors, GhMYB7 and GhMYBL1, play a role in the formation of fiber SCW. *GhMYB7* regulates cotton fiber SCW cellulose synthase by directly binding to three different *cis*-elements in the *GhCesA4*, *GhCesA7*, and *GhCesA8* promoters. Overexpression of *GhMYB7* increases cell wall thickness (Huang et al., 2016, 2021b). In *GhMYBL1* transgenic plants, the biosynthesis of cellulose and lignin is enhanced compared to in the wild type (Sun et al., 2015b). Therefore, *GhMYB7* and *GhMYBL1* may be involved in the regulation of cotton fiber SCW biosynthesis and deposition. The lipase/hydrolase gene *GhGDSL* from *Gossypium hirsutum* is expressed during SCW biosynthesis (19–25 DPA), and *GhMYB1* is specifically expressed in 19 DPA fibers. A yeast one-hybrid assay showed that *GhMYB1* binds to the *GhGDSL* promoter, indicating that *GhMYB1* can regulate *GhGDSL* during fiber development (Yadav et al., 2017).

The CELLULOSE SYNTHASE A (*GhCESA*) genes in cotton are divided into six subclasses, of which *GhCESA4, GhCESA7*, and *GhCESA8* are mainly involved in SCW development (Yuan et al., 2015a; Wang et al., 2019b; Zhang et al., 2021e). *GhCESA4* positively regulates cellulose biosynthesis during cotton fiber development (reviewed by Xu et al., 2021). As the developing cotton fiber produces SCW cellulose, the transcription level of *GhCESA4* is significantly up-regulated (Kim et al., 2011).

*GhTCP4* contains a conserved non-standard bHLH domain. During fiber development, transcriptome and promoter activity analysis showed that the overexpression of *GhTCP4* up-regulates and accelerates SCW biosynthesis in fiber cells. The activation of the pathway leads to shorter fibers, different lengths, and thicker walls (Cao et al., 2020; Huang et al., 2021a). GhTCP4 interacts with GhHOX3 and inhibits its transcriptional activity. *GhTCP4* and *GhHOX3* antagonistically regulate cell elongation, thereby establishing the time control of the transition of fibroblasts to SCW (Ye et al., 2020; Huang et al., 2021a). *GhTCP14* is also a key gene in the AUX pathway (Wang et al., 2013a) and promotes the biosynthesis of GA (Hao et al., 2012). The E3 ligase GhHUB2 can control fiber development by triggering the accumulation of GhKNL1. Overexpression of *GhHUB2* can increase cotton fiber length and secondary cell wall thickness. The transcription inhibitor *GhKNL1* is mainly expressed in developing fibers and can be degraded by *GhHUB2* through the ubiquitin-26S proteasome pathway, eliminating the inhibitory effect of *GhKNL1* on the accumulation of cotton fiber SCWs (Feng et al., 2018; Hao et al., 2018). GhKNL1 regulates fiber elongation and SCW formation, and can directly bind to the promoters of *GhCesA4-2/4-4/8-2* and *GhMYB46* to regulate cellulose biosynthesis during SCW deposition. *GhKNL1* can also inhibit the expression of *GhXPA2D/4A-1/4D-1/13A* by combining with the promoter of *GhXPA2D/4A-1/4D-1/13A*, thereby regulating the fiber elongation of cotton (Wang et al., 2021d).

During the cotton fiber maturation stage (40–50 DPA), overexpression of the marshmallow sucrose synthase gene *GhSusA1* can significantly increase the length and thickness of mature fibers. Overexpression of *GhSusA1* can enhance secondary cell wall thickening and fiber quality compared to that in the wild type; whereas inhibition of *GhSusA1* in transgenic cotton reduces fiber quality. Exogenous application of bioactive GA can promote the expression of *GhSusA1* in cultured fibers and cotton hypocotyls (Jiang et al., 2012; Bai et al., 2014; Wang et al., 2020a). *GhBRI1* is a BR receptor that regulates cellulose deposition during the SCW deposition and fiber maturation stages (Li et al., 2011; Sun et al., 2015c).

**FIGURE 2:**
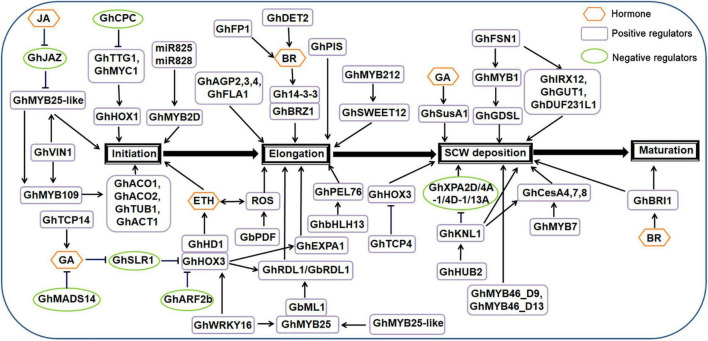
Regulatory network model for trichome (fiber) development in cotton.

A regulation model of cotton trichome (fiber) initiation, elongation, secondary cell wall deposition and maturation are shown in Figure 2.

## Molecular Mechanisms of Bicellular Trichome Development

### Trichome Development in *Oryza sativa*

Rice (*Oryza sativa*) leaf trichomes are bicellular. They are arranged in a cylindrical shape between the stem veins of the upper and lower epidermis of the leaves, the morphological photographs of rice leaf trichomes were shown in Supplementary Figure 2 (Wang et al., 2013b). Rice trichomes commonly occur on the surface of leaves and glumes. They can be classified as macrohairs, micro hairs and glandular hairs (Wang et al., 2013b). Macrohairs and micro hairs are ubiquitous in plants. Macrohairs are mainly distributed on siliceous cells of cell vascular bundles, while micro hairs and glandular hairs are mainly distributed in the vicinity of stomata or motile cells (Wang et al., 2013b). Rice light-leaf mutants have filamentous glandular hairs that release secretions to the epidermal surface (Li et al., 2010a). Rice trichome development is a very complex process. Although some rice trichome-related genes have been located and cloned, the molecular mechanisms of rice trichome development are still largely unknown. At present, few genes involved in rice trichome development have been identified in the fate determination, initiation and elongation stages.

Several genes encoding different types of proteins and transcription factors are involved in the fate determination and initiation stage.

ESTRICTION FRAGMENT LENGTH POLYMORPHISM (RFLP) marker was used to preliminarily map *GL-1*, which controls the light-leaf trait of tropical japonica rice, on chromosome 5, which was linked to RG182 and RG403 (Yu et al., 1995). Researchers constructed the near isogenic line (NIL) of light-leaf rice using the American rice variety Rico No. 1 and light-leaf rice variety Jia64 (Li et al., 2012a). The light-leaf gene *GLABROUS RICE1* (*GLR1*) was located within 21 kb between chromosome 5 markers M6 and M7. In a complementary experiment, weakening the expression of *GLR1* significantly reduced or completely removed the number of trichomes, and the constitutive expression of *GLR1* in the knockout line *NILglr1* rescued the hairless phenotype of T0 generation transgenic lines (Li et al., 2012a). Complementary tests showed that the genomic fragments covering the open reading frame of *DEGENERATIVE PALEA* (*DEP*) can restore the formation of bristle-type trichomes on the leaves and glumes of hairless rice, indicating that *DEP* can regulate trichome formation on leaves and glumes (Angeles-Shim et al., 2012). *GLR1*, *WUSCHEL-LIKE HOMEOBOX 3B* (*OsWOX3B*), *DEP* and *NUDA* encode a homologous domain protein similar to that encoded by *WUSCHEL* (*WUS*); these genes are located at the same site on chromosome 5 and regulate rice trichome formation (Shang et al., 2020; Li et al., 2021a). The *NUDA* and *GL-1* genes are alleles, and RNAi and complementary transgene studies have shown that this locus encodes the WUSCHEL-like homeobox gene *OsWOX3B* (Zhang et al., 2012). *GLR2* has been screened from the tissue seedlings of Zhonghua 11, a light-leaf mutant. Genetic analysis revealed that *glr2* mutants are controlled by a single-site recessive gene. A segregating population was used to locate *glr2* on the first chromosome markers RM12124 and RM12136. There are 12 predicted genes in the 84.7 kb interval between the two genes, and the expression of three genes changed significantly, of which the expression of two genes (*LOC_Os01g70020* and *LOC_Os01g70090*) was induced in the light-leaf mutant and that of one gene (*LOC_Os01g70100*) was repressed in the *optica* mutant. Therefore, *LOC_Os01g70100* is a candidate gene for *GLR2* (Wang et al., 2013b).

The recently reported SQUAMOSA PROMOTER BINDING PROTEIN BOX (SBP Box) family gene *SQUAMOSA PROMOTER BINDING PROTEIN-LIKE10* (*OsSPL10*) also controls trichome initiation and positively regulates rice trichome formation (Tao et al., 2019). Knockout *OsSPL10* rice displayed glabrous leaves and glumes, whereas overexpression mutants exhibited the opposite phenotype (Tao et al., 2019). At the same time, the researchers also found that *OsSPL10* transcript levels increased when treated with the auxin analog 1-naphthylacetic acid (NAA) (Li et al., 2021a). *OsTCL1* is a homolog of *Arabidopsis AtTCL1*, and *OsTCL1* transgenic lines in *Arabidopsis* inhibit the formation of trichomes through direct interaction with *GL3*. However, there is no phenotypic difference in *OsTCL1* overexpressing rice plants (Zheng et al., 2016). HAIRY LEAF6 (OsHL6) encodes APETALA2/ETHYLENE RESPONSE FACTOR type transcription factor. OsHL6 protein can interact with *OsWOX3B*, and enhance the binding ability of *OsHL6* to the downstream AUX-related gene *OsYUCCA5* (Sun et al., 2017).

*OsGL6* localizes to the lower 79-kb region of chromosome 6. *OsGL6* is a natural allele of *OsHL6*, which can stimulate the initiation of trichomes. In *OsGL6* overexpressing rice, *OsGL6* can promote trichome initiation but does not affect trichome elongation (Xie et al., 2020b). Furthermore, *OsGL6* can interact with OSK3 and CSN5 proteins, and *OsGL6* may regulate the formation of trichomes by forming a complex with OSK3 and CSN5 (Xie et al., 2020b).

In the rice trichome elongation stage, *OsHL6* can not only promote trichome initiation but also affect rice trichome elongation. Suwangwanger (SWWR) is an indica rice variety from Sri Lanka with hairy leaves. *HL6^SWWR^* is a novel allele of the reported HL6 locus that regulates trichome formation in rice. Transgene complementation and knockout experiments confirmed that *HL6^SWWR^* regulates rice trichome elongation (Fei et al., 2020).

**FIGURE 3:**
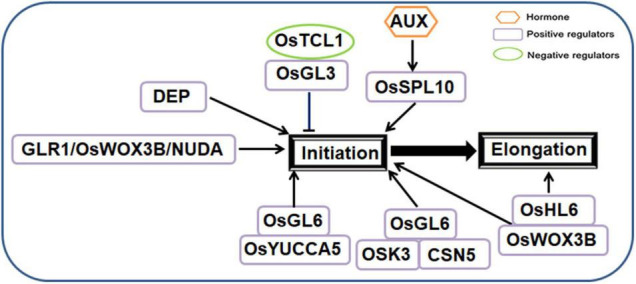
Regulatory network model for trichome development in rice.

A regulation model of rice trichome initiation and elongation is shown in Figure 3.

## Molecular Mechanisms of Multicellular Trichome Development

### Trichome Development in *Cucumis sativus*

Cucumber (*Cucumis sativus*) is a common vegetable worldwide. From stems and leaves to flowers, branches, fruits, and tendrils, their surface is covered with trichomes. The trichomes on its fruit are called thorns, and they have a multi-layered tubercule underneath (Pan et al., 2015). Both the tubercule and the thorns are collectively referred to as cucumber fruit wart. In cucumber, thorns combine with tubercule to form a verrucous fruit trait, which is important for fruit quality (reviewed by Liu et al., 2016).

Cucumber fruit spines are multicellular trichomes, and their development and regulation mechanisms are different from those of unicellular trichomes. According to the structure and morphology of cucumber fruit trichomes, the researchers divided cucumber fruit trichomes into eight types, the morphological photographs of cucumber trichomes were shown in Supplementary Figure 3 (Xue et al., 2019). Of these, type I and VI trichomes are glandular hairs (Xue et al., 2019). Except for two hairless mutants (*csgl1* and *csgl3*), type I trichomes exist on the fruits of all cucumber varieties. Type I trichomes consist of a single short stalk with 3–5 cells and a 4–8-cell head region with glandular functions (Samuels et al., 1993). Type II trichomes are larger, without glands, and consist of a base and long stems. During non-glandular hair type II trichome development, precursor cells expand perpendicular to the surface of the epidermis and then divide around the cells to form a stalk composed of 5–7 rectangular cells and a pointed apical cell (Li et al., 2015). The stalk of type III trichomes consists of 3–6 cells and is much shorter than the stalk of type II trichomes. A particularly notable feature of type III trichomes is that the base cells divide to form a conical structure. Type IV trichomes undergo initiation and division to form multicellular stalks, and the base cells divide into multicellular structures similar to type II trichomes but smaller. Type V trichomes have a unique pyramid structure without an obvious slender stem and without an enlarged base (Xue et al., 2019). Type VI glandular hairs are rarely observed and have similar four-cell or five-cell glands on each head, but their stalk cells are longer than those of type I trichomes. Type VII and VIII trichomes are only found on the hairless mutant *csgl1*; they are invisible to the naked eye (Chen et al., 2014; Zhao et al., 2015). Type VII trichomes begin with the expansion of a hairy cell. Then, after several cell divisions, multiple cells are stacked together to form a blunt tip. The development process of type VIII trichomes is basically similar to that of type VII trichomes, but type VIII trichomes have extra branch formation in the later stage (Zhao et al., 2015). All non-glandular trichomes have gone through a stage of senescence, that is, the tip first becomes white or brown and then the other trichomes lose their green color from top to bottom and become white or brown (Zhang et al., 2016). The trichomes of cucumber cotyledons and fruits have relatively synchronized developmental trajectories and can be divided into five stages according to their morphogenesis: initiation, first division, formation of pointed head (non-glandular trichomes)/glandular head transformation (glandular trichomes), elongation (non-glandular trichomes)/glandular head formation (glandular trichomes) and multicellular base formation (non-glandular trichomes)/active metabolic processes (glandular trichomes) (Dong et al., 2022).

HD- ZIP-, MYB-, and C2H2-type transcription factors as well as WD-repeat proteins are involved in the cucumber trichome fate determination and initiation stage.

*TRICHOME-LESS (Tril)* and *CsGL3* are two alleles of the HD-ZIP IV family; they play an important role in the fate determination and initiation of cucumber glandular hairs. Mutations in these two genes present a completely hairless phenotype on leaves, stems, flowers, sepals, and fruits (Pan et al., 2015; Wang et al., 2016). *Tril* expression controlled by its own promoter partially rescues the mutant phenotype of *tril* and *csgl3* (Du et al., 2020). The hairless phenotype of *csgl3* exhibited due to the loss of function of *CsGL3* is due to the insertion of an autonomous, active class I transposable element in *CsGL3* (Pan et al., 2015). It is speculated that *Tril* and *CsGL3* may directly bind to *CsTBH*, *CsMICT*, and *CsGL1* to activate downstream transcriptional activators such as *CsMYB6*, *CsWIN1*, and *CsGL2*, and then promote the initiation of trichomes in leaves, stems and fruits (Pan et al., 2015; Shvachko et al., 2020). The *csgl2* mutant showed few trichomes or nodules on tendrils, calyx, ovary and fruit, but glabrous stems and leaves (Cui et al., 2016).

*GLABROUS 1* (*CsGL1*), *TINY BRANCHED HAIR* (*CsTBH*), and *MICRO-TRICHOME* (*CsMICT*) genes of the HD-ZIP I family are involved in the formation of cucumber trichomes. Under scanning electron microscopy (SEM), many papillae can be observed on the epidermis of *csgl1* mutant leaves. The density of papillae is similar to that of wild type trichomes, indicating that *CsGL1* may be involved in the development of trichomes of leaves but not in fate determination and initiation (Pan et al., 2015). Studies have shown that CsGL1 may indirectly regulate the expression of *CsMYB6* and *GA20ox1* (Li et al., 2015). The other two allelic mutants of *csgl1*, *tbh*, and *mict* have similar phenotypes to *csgl1*. *CsTBH* is preferentially expressed in the multicellular trichomes of cucumber fruits. Overexpression of *CsTBH* in *cstbh* mutants restores the fruit trichome phenotype, while the silence of *CsTBH* in wild-type plants leads to spine dysplasia; however, *CsTBH* does not participate in the spine initiation (Chen et al., 2014; Xue et al., 2019). *CsTBH* can directly bind to the promoter of the cucumber 1-aminocyclopropane-1-carboxylic acid synthase (*CsACS*) gene and regulate its expression, thereby affecting the development of multicellular fruit trichomes (Zhang et al., 2021f). Therefore, *CsTBH* regulates cucumber fruit trichomes through the ETH pathway (Chen et al., 2014; Xue et al., 2019). *CsMICT* is expressed in trichome cells, and all leaf and fruit trichomes in its mutants are small and stunted (Zhao et al., 2015; Pan et al., 2021). Phenotypic analysis of mutants demonstrated that *CsTBH*, *CsMICT* and *CsGL1* are involved in regulating the morphogenesis of glandular hairs rather than fate determination and initiation (Liu et al., 2016; Feng et al., 2021b).

The MYB-type transcription factors CsMYB6 (Csa3G824850) and CsTRY (Csa5G139610) negatively regulate cucumber fruit spine initiation (Zhang et al., 2019b). Transformation of *CsTRY* (*Csa015371*) into *Arabidopsis* can significantly inhibit the development of leaf trichomes. Researchers speculate that the *CsTRY* gene inhibits the development of cucumber trichomes, and the *CsMYB6* gene, which is similar to *CsTRY*, negatively regulates the formation of cucumber trichomes (Yang et al., 2018, 2021).

The C2H2 zinc finger protein gene *Tu* is specifically expressed in cucumber tumor cells (Yang et al., 2014a). *Tu* is a key factor in controlling fruit tumor formation (Cui et al., 2016). *CsGL1* has an epistatic effect on *Tu* (reviewed by Che and Zhang, 2019). The formation of nodules is related to high CTK content, and the expression of *Tu* affects the expression of *Csa5M644580* and *Csa5M224130* genes, which are homologous to CTK hydroxylase, thereby accelerating the biosynthesis of CTK and promoting the formation of fruit nodules (Yang et al., 2014a). *Tu* was present in all 38 warty (Wty) lines and completely absent in all 56 non-wart (nWty) lines. Moreover, *Tu* was required for the Wty fruit phenotype in *Tu* transgenic cucumber plants (Yang et al., 2014a).

The WD-repeat protein gene *CsTTG1* (*Csa4Gm097650*) plays a key role in cucumber flowering and trichome and fruit tumor formation. Silencing of *CsTTG1* inhibits the formation of fruit thorns (Guo et al., 2020). Molecular and genetic analysis showed that *CsTTG1* and the key trichome forming factors *CsMICT* and *CsGL1* have similar roles in regulating the initial development of trichomes (Chen et al., 2016a).

The bHLH-type transcription factor HECATE2 (CsHEC2) is highly expressed in cucumber pericarp (including tubercule and thorns). A mutant of the *CsHEC2* gene obtained using the CRISPR/Cas9 system had a significantly lower fruit thorn and tumor density and lower accumulation of CTK compared to the wild type. Conversely, overexpression of *CsHEC2* led to an increase in thorn and tumor density and CTK level. CsHEC2 directly binds to the promoter of the cytokinin hydroxylase gene *CsCHL1*, activating its expression. In addition, CsHEC2 directly interacted with CsGL3 and Tu, further enhancing its positive regulation of CsCHL1 expression (Wang et al., 2021e).

The plant hormone signaling pathway is also involved in cucumber trichomes occurrence. 6-benzylaminopurine (6-BA) and GA3 effect epidermal differentiation and fruit thorn development. They affect the number of trichomes in each fruit. Both 6-Benzylaminopurine (6-BAP) and GA stimulate the formation of trichomes in cucumber fruit in a concentration-dependent manner (Zhao et al., 2015; Xue et al., 2019). GA oxidase (GAoxs) is a key enzyme in the GA biosynthesis pathway. Overexpression of *CsGA20ox1* reduces the length of cucumber fruit spines (Li et al., 2015; Sun et al., 2018). The *NUMEROUS SPINES* (*NS*) (*Csa2G264590*) gene encodes AUX transporter-like protein 3, which is a key negative regulator gene that determines the thorn density of cucumber fruit (Du et al., 2020). Through expression pattern analysis, it was found that the upstream genes of the AUX signaling pathway in two cucumber cultivars (NCG122 and NCG121), including *NS*, were down-regulated, while AUX signaling pathway downstream genes were up-regulated. This implies that *NS* is a negative regulator that determines the density of cucumber fruit thorns and may regulate the development of fruit thorns by regulating the AUX signaling pathway (Xie et al., 2018).

**FIGURE 4:**
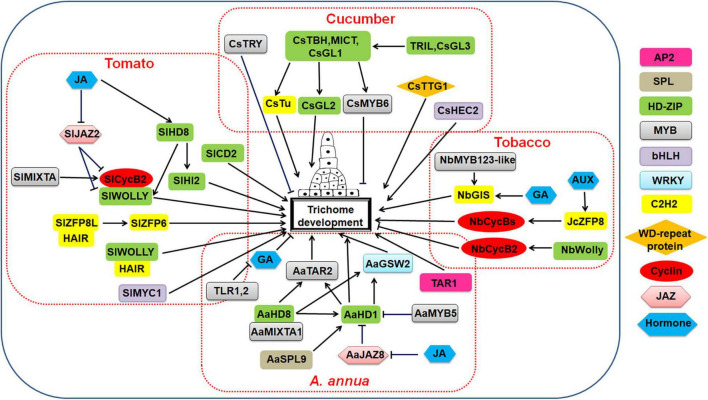
Regulatory network model for multicellular trichome development in cucumber, tomato, tobacco, and *Artemisia annua*.

A regulation model of cucumber trichome development was shown in Figure 4.

### Trichome Development in Tobacco

Tobacco (*Nicotiana tabacum* L.), including *Nicotiana benthamiana* L., is commonly used as a heterogeneous production platform as an important model plant for basic biological research. It is a multicellular hairy plant that is widely cultivated worldwide. Most studies on multicellular trichomes in tobacco have primarily focused on morphological observations and identification of their secretions; few studies have investigated the molecular mechanisms of multicellular trichome differentiation in tobacco. Tobacco has three types of trichomes: long-stalked glandular trichomes (LGT), short-stalked glandular trichomes (SGT) and non-glandular trichomes (NGT) (Wang et al., 2021f; Zhang et al., 2021a). The glandular trichomes consist of one basal cell, 1–5 stalk cells and 1–12 head cells. They are mainly unbranched head-shaped glandular hairs composed of a multicellular stalk and a single or multicellular head, the morphological photographs of tobacco trichomes were shown in Supplementary Figure 4 (Amme et al., 2005; Liu et al., 2018a).

The C2H2 transcription factor is involved in regulating the development of tobacco glandular trichomes. *NbGIS* has a positive regulatory effect on the development of tobacco glandular trichomes. In response to GA signals, it can control glandular trichome initiation and significantly affects the accumulation and expression of GA biosynthesis marker genes, which may lead to tobacco growth and maturity (Liu et al., 2018b). The *Jatropha curcas* C2H2 zinc finger protein gene, *JcZFP8*, regulates the development of transgenic tobacco trichomes (Li et al., 2016). *JcZFP8* is related to *NtZFP8*, *AtGIS* and *AtZFP8* during trichome development (Liu et al., 2017). Overexpression of JcZFP8 in tobacco increases the number of trichomes on flowers, and JcZFP8 induces trichome formation. The way *JcZFP8* regulates trichome development is different from that of *GIS*. *JcZFP8* regulates trichome development by inducing the expression of *MYB* and *CycB* related genes. This finding provided new insights into the regulatory mechanism of the C2H2 ZFP genes in trichome development (Shi et al., 2018).

MYB-type transcription factors play a role in tobacco trichome initiation (Schnittger et al., 2005). *NbMYB123-like* encodes an R2R3 MYB domain that regulates tobacco glandular trichome initiation by acting downstream of *NbGIS* (Liu et al., 2018b). The R2R3 MYB transcription factors MIXTA and MIXTA-like1 from snapdragon promote tobacco trichome development (Perez-Rodriguez et al., 2005).

The interaction between HD-ZIP transcription factors and B type cyclin genes regulates tobacco trichome development. The tobacco B type cyclin gene *NbCycB2* is a negative regulator of multicellular trichome formation, and the *NbWoolly* (*Nbwo*) gene is an HD Zip IV transcription factor (Yang et al., 2015). *NbWoV* is a *Nbwo* gain-of-function allele (Wu et al., 2020b). The genomic sequences of *Nbwo* and *NbWoV* can be combined with the *NbCycB2* promoter sequence to directly regulate the expression of *NbCycB2*, *Nbwo* and *NbWoV*. As a form of feedback regulation, at the protein level, *NbCycB2* inhibits Nbwo activity to negatively regulate trichome formation. Mutation of the *Nbwo* wolly motif prevents *NbCycB2* inhibition of *NbWoV*, resulting in a marked increase in the amount of active Nbwo protein, increasing trichome density and branching number (Wu et al., 2020b). *Nbwo* and *NbWoV* act on the L1-like box in the promoter region of *NbCycB2* to affect the expression of *NbCycB2*, inhibit the activity of *Nbwo*, and regulate the level of proteins that form trichomes (Wu et al., 2020b). In addition, *NbCycB2* can inhibit trichome initiation by binding to the LZ domain of *NbWo66* (Wu et al., 2020b). Knocking out *NtCycB2* (*NtCycB2-KO*) can promote the formation of LGT, while overexpression of *NtCycB2* (*NtCycB2-OE*) can reduce LGT density (Wang et al., 2021f).

Various plant hormones are involved in tobacco trichome formation and branching (Di et al., 2015). In *JcZFP8* transgenic tobacco, AUX may be involved in trichome initiation, while GA and JA may not be involved in this process, and SA is a negative regulator of trichome development (Traw and Bergelson, 2003; Han et al., 2021). The tobacco transcription repressor protein NtJAZ is potentially involved in abiotic stress responses and glandular hair development. *NtJAZ-9* may play an important role in the induction of *NtJAZs* in glandular trichomes (Zhang et al., 2019a).

A regulation model of tobacco trichome development was shown in Figure 4.

### Trichome Development in Tomato

Cultivated tomato (*Solanum lycopersicum*) and its wild relative (*Lycopersicon esculentum*) have seven different trichome types. Types II, III, and V are non-glandular hairs composed of a neck and base. They act as a physical barrier to function in resisting diseases and insects. Types I, IV, VI, and VII are glandular hairs, the morphological photographs of tomato trichomes were shown in Supplementary Figure 5 (Xu et al., 2018; Chalvin et al., 2020). In addition to the neck and base, they also have a gland top that can store and secrete various secondary metabolites (Chen et al., 2018). Type II and V tomato trichomes are the most abundant; they are the typical non-glandular hairs. Type II and V trichomes have similar morphology but are composed of different numbers of cells. Type II trichomes are mainly composed of 5–8 cells, and type V trichomes are mainly composed of three cells. In type II trichomes, cells are larger at the base and become smaller toward the top. A similar trend occurs in type V trichomes (Glas et al., 2012; Vendemiatti et al., 2017). Type III trichomes are shorter than type II trichomes. Most type I and VI trichomes are glandular. Type I trichomes have multicellular bases, multicellular stalks and glandular cells at the top, whereas type VI trichomes have shorter multicellular stalks and a pumpkin-shaped gland composed of four cells (Bergau et al., 2015). Type IV trichomes have glands similar to type I trichomes, with a single-cell base and two to three stalk cells, whereas type VII trichomes have shorter single-cell stalks and 4–8-cell glands (Vendemiatti et al., 2017).

Tomato trichomes are mainly regulated by R2R3-MYB, HD-ZIP IV, C2H2, and bHLH-type transcription factors (reviewed by Chezem and Clay, 2016; see also Chalvin et al., 2020).

The R2R3-MYB proteins SlMIXTA1 and SlMixta-like control tomato trichome initiation. After *SlMixta-like* silencing, many trichomes occur on leaves. *SlMixta-like* silence lines lead to abnormal trichome spacing and trichome aggregation (Ewas et al., 2017; Galdon-Armero et al., 2020). In *SlMixta-like* overexpression lines, the density of type I and IV trichomes was unaffected, while the density of type V and VI trichomes was significantly reduced compared to that in the wild type (Galdon-Armero et al., 2020).

HD-ZIP IV transcription factors are key regulators of tomato trichome development (Hua et al., 2021a). The *SlWOOLLY* (*SlWo*) gene encodes an HD-ZIP IV transcription factor that regulates tomato type I trichome formation mainly through heterodimer formation with the B-type cyclin *SlCycB2 (Gao et al., 2015)*. *SlWo* also actively regulates mitosis in multicellular trichomes (Gao et al., 2017; Hua et al., 2021a). *SlWo* is mainly regulated by plant hormones such as JA, AUX and GA. Among them, JA has an obvious effect on inducing glandular hairs. The HD-ZIP IV transcription factor SlCD2 regulates type VI glandular trichome formation (Nadakuduti et al., 2012). A loss-of-function mutation in *SlCD2* resulted in a sticky peel mutant phenotype in tomato, with less glandular hairs (especially type VI) than that in the wild type (Nadakuduti et al., 2012). Knockout of the HD Zip IV transcription factor HDZIPIV8 also distorts tomato trichomes (Xie et al., 2020a). A mutation of *NCK-ASSOCIATED PROTEIN 1* named as *Hairless-2* (*Hl-2*) in tomato caused serious distortions in all trichome types (Xie et al., 2020a). *HDZIPIV8* regulates the expression of *H1-2* by binding to the L1-Box of the *H1-2* promoter region, thereby regulating the elongation and morphogenesis of tomato trichomes (Xie et al., 2020a). *SlHZ45* is the HD-ZIP IV transcription factor with the highest homology to *SlWo*. Compared with a control group, the number of type I, IV and VI trichomes on the leaf margins of *SlHZ45* overexpressing plants increased; these three trichome types are glandular hairs (Zhang et al., 2014). The HD-Zip transcription factor SlHD8 positively regulates tomato trichome elongation. Dual luciferase and ChIP experiments showed that *SlHD8* regulates tomato trichome elongation by directly binding to a set of cell wall loose protein gene promoters and activating their transcription (Hua et al., 2021b).

C2H2 zinc finger proteins have been isolated and identified in tomato trichome formation. The C2H2 zinc finger protein gene is homologous with *ZFP8* of *Arabidopsis*. It directly interacts with *SlWo* and regulates type I and VI trichomes (Chang et al., 2018). *H* overexpression lines can promote the elongation of type I, III and VI trichomes in tomato. *H* is a constitutively expressed gene, and lack of *H* may inhibit the function of *SlWo* (Tao et al., 2014). In addition, the similar regulatory effects of *H* gene homologs in the formation of trichomes indicate that these multicellular structures of solanaceous plants may be controlled by conservative molecular mechanisms. Recent studies have found that *SlZFP8-like* (*SlZFP8L*) directly interacts with *SlWo* to regulate tomato trichome initiation (Zheng et al., 2021). Overexpression of *SlZFP8L* will increase the length of type I, III, and VI trichomes and increase the density of type I, III, V, VI, and VII trichomes (Zheng et al., 2021). Researchers also found that *H* interacts with *SlZFP8-like* (*SlZFP8L*) to regulate trichome initiation and elongation by regulating *SlZFP6* expression in tomato (Zheng et al., 2021).

Almost all genes identified so far that control glandular hair also affect non-glandular hair development; only one gene (*SlMYC1*) is involved in glandular hair regulation but not non-glandular hair regulation. SlMYC1 is a bHLH transcription factor that regulates the development of type VI glandular hairs in tomato (Xu et al., 2018). In mature gland cells of type VI trichomes, *SlMYC1* can be recruited by *SlWo* to activate TPS gene expression for terpene biosynthesis. The SlWo-SlMYC1 functional module can be inhibited by SlJAZ2, and JA can relieve this inhibition. In addition to acting with SlWo, SlMYC1 plays a SlWo-independent role in glandular cell division and expansion (Xu et al., 2018). *SlbHLH95* negatively regulates trichome initiation, and plants overexpressing *SlbHLH95* have significantly less type I trichomes on stems (Chen et al., 2020). *bHLH95* also regulates GA biosynthesis and trichome formation through *GA20ox2* and *KS5* (Chen et al., 2020).

Plant hormone signaling pathways are involved in regulating tomato trichome differentiation. The JAZ protein *SlJAZ2* is a repressor protein of the tomato JA signaling pathway; it also negatively regulates the occurrence of glandular hairs. In *SlJAZ2* overexpression plants, the expression of *SlWo* and *SlCycB2* was suppressed, indicating that *SlJAZ2* suppressed glandular hair initiation by suppressing the expression of *SlWo* and *SlCycB2* (Yu et al., 2018). A yeast two-hybrid experiment found that SlJAZ2 interacts with CORONATINE INSENSITIVE1 (SlCOI1), which also positively regulates glandular hair development (Thines et al., 2007). AUX also plays an important role in regulating glandular hair growth. When the expression of the tomato AUX family gene *SlIAA15* and the AUX response factor *SlARF3* is down-regulated, the number of tomato type I, V, and VI trichomes decreases (Deng et al., 2012b). The negative regulator of trichome development *JAZ4* is a key component of tomato trichome JA signal transduction. The HD-Zip transcription factor SlHD8, located downstream of the JA signal, positively regulates tomato trichome elongation and can interact with *SlJAZ4* (Hua et al., 2021b).

A regulation model of tomato trichome development was shown in Figure 4.

### Trichome Development in *Artemisia annua*

In recent years, artemisinin has received widespread attention due to its antimalarial effects. The glandular hair of *Artemisia annua* is the only place where artemisinin is synthesized and stored. *Artemisia annua* has two types of trichomes, namely non-secretory glandular trichomes and glandular secretory trichomes (GSTs). Non-secretory glandular trichomes are also called T-shaped trichomes (TSTs) or T-type non-glandular trichome (TNGTs) because their appearance is similar to the letter “T.” The morphological photographs of *Artemisia* trichomes were shown in Supplementary Figure 6 (Chalvin et al., 2020).

The transcription factors involved in the regulation of glandular hair development in *A. annua* are similar to those in tomato, and most of them belong to the R2R3-MYB and HD-ZIP subfamilies (reviewed by Chezem and Clay, 2016; see also Chalvin et al., 2020).

*AaHD1* and *AaHD8* of the HD-ZIP IV family play a positive role in regulating the initiation of glandular hairs. Overexpression of *AaHD1* or *AaHD8* increases the density of glandular hairs, whereas inhibition of expression of either of them decreases the density of glandular hairs. SEM analysis found that the glandular and non-glandular hair densities of *A. annua* were significantly decreased in *AaHD8* silenced lines and increased in *AaHD8* overexpressing lines compared with the wild type. In addition, expression levels of *AaHD1* followed changes in *AaHD8* expression, which was significantly down-regulated in *AaHD8* silencing and up-regulated in *AaHD8* overexpressing *A. annua* lines (Yan et al., 2018).

The R2R3-MYB transcription factor AaMIXTA1 is a positive regulator of the initiation of glandular hair development and can directly activate the expression of genes related to the biosynthesis of the leaf stratum corneum (Matías-Hernández et al., 2017). Later research found that the transcription complex formed by *AaHD8* and *AaMIXTA1* can promote *AaHD1* expression, which in turn promotes glandular hair initiation (Yan et al., 2018). Overexpression of *AaMYB1* can increase the density of glandular hairs (Matías-Hernández et al., 2017). *AaMYB1* is an ortholog of *AtMYB61*. Overexpression of both *AaMYB1* and *AtMYB61* affect *Arabidopsis* trichome initiation, root development, and stomatal pore size. Transgenic *Arabidopsis* overexpressing *AaMYB1* have a higher density of trichomes on rosette leaves compared to the wild type, and a similar number of trichomes as *atmyb61-2* mutant plants overexpressing *AaMYB1* (*myb61-2* 35S:*AaMYB1*), indicating that *AaMYB1* expression rescues the *Atmyb61-2* mutant phenotype (Matías-Hernández et al., 2017). *AaMYB16* positively regulates the initiation of glandular hairs. Compared with the wild type, *AaMYB16*-overexpressing transgenic lines had 28–45% higher GST density on the front surface of mature leaves, while *AaMYB16*-knockdown lines had 27–41% lower GST density (Xie et al., 2021a). SEM analysis showed that TNGT density was also higher in the overexpression lines and lower in the RNAi lines (Xie et al., 2021a). *AaMYB5* has the opposite effect to *AaMYB16* but neither can independently regulate GST formation. GST densities were higher in *AaMYB5* knockdown lines and lower in *AaMYB5* overexpression lines compared to the wild type. SEM analysis showed no difference in TST density between wild type and transgenic plants (Xie et al., 2021a). *AaMYB5* and *AaMYB16* compete by interacting and regulating the binding activity of the *AaHD1* promoter (Xie et al., 2021a). R2R3 MYB transcription factor AaTAR2 is mainly expressed in young leaves of *A. annua*. Knockout and overexpression of *AaTAR2* resulted in decreased and increased GST number and artemisinin content, respectively. In addition, the researchers also found that AaTAR2 can bind to the promoters of HD-ZIP transcription factors AaHD1 and AaHD8 to regulate the development of *Artemisia* trichomes (Zhou et al., 2020). R2R3 MYB transcription factors TRICHOME LESS REGULATOR 1 (TLR1) and TLR2 were found to negatively regulate trichome development in *A. annua*, the trichome density and artemisinin content were decreased in *TLR1* and *TLR2* overexpressing lines, and increased in *TLR1*-RNAi lines compared with the wild type. Studies of *TLR1* and *TLR2* in *Arabidopsis* and *A. annua* overexpression lines show that they negatively regulate trichome density by reducing gibberellin levels (Lv et al., 2022).

Overexpression of the GST-specific WRKY transcription factor AaGSW2 in *A. annua* significantly increases GST density. Knockout of *AaGSW2* inhibits the initiation of GST in *A. annua*. Furthermore, *AaHD1* and *AaHD8* can bind to the L1-box on the promoter of *AaGSW2* and positively regulate GST initiation (Xie et al., 2021b).

The AP2 transcription factor TRICHOME AND ARTEMISININ REGULATOR 1 (TAR1) regulates trichome development in *A. annua*. Compared to the wild type, *TAR1* disturbed expression lines had altered trichome morphology and cuticle wax composition; significantly lower artemisinin content; higher permeability; less glandular hairs; and a collapsed glandular hair head phenotype (Tan et al., 2015). In addition, compared to the wild type, *A. annua* plants overexpressing the β-glucosidase gene (*BGL1*) had 20 and 66% more trichomes with 1.4 and 2.56% higher artemisinin content on leaves and inflorescences, respectively (Singh et al., 2016).

SQUAMOSA promoter-binding protein-like (SPL) is a kind of plant-specific transcription factor, studies have shown that overexpression of *AaSPL9* increases the density of glandular hairs by 45–60% and the artemisinin content by 33–60%, indicating that *AaSPL9* positively regulates the initiation of glandular hairs. Yeast one-hybrid, dual-luciferase and electrophoretic mobility shift assay (EMSA) demonstrated that AaSPL9 activates the expression of *AaHD1* by directly binding the GTAC-box of the *AaHD1* promoter to regulate the initiation of glandular hairs in *A. annua* (He et al., 2022).

The plant hormone signaling pathway is involved in *A. annua* trichome initiation. Trichome formation is mainly regulated by JA. *AaJAZ8* is a repressor of the JA signaling pathway in *A. annua*. It can inhibit the activity of the positive regulator *AaHD1* and reduce the density of glandular hairs (Yan et al., 2017). The zinc finger protein *AaSAP1* can also respond to JA induction and positively regulate glandular hair development. However, the specific molecular mechanisms remain to be elucidated (Wang et al., 2019c).

A regulation model of *A. annua* trichome development was shown in Figure 4.

## Molecular Mechanisms of Trichome Development in Other Plants

Three types of trichomes occur on adult maize (*Zea mays*) leaves: microscopic bicellular hairs, macrohairs, and prickle hairs (Kong et al., 2021). Overexpression of the maize HD-ZIP IV gene *OCL4* in *Arabidopsis* resulted in a glabrous rosette leaf phenotype (Vernoud et al., 2009). The maize *GLOSSY1* (*ZmGL1*) gene is a component of the pathway leading to maize seedling epidermal wax biosynthesis. *zmgl1* mutants have altered maize epidermal development, including altered trichome size and impaired cuticle structure (Sturaro et al., 2005). The SQUAMOSA-promoter-binding protein-like (SPL) protein is a plant-specific transcription factor with SBP characteristics and a domain consisting of 76 amino acid residues (Wei et al., 2018a). Cytological analysis showed that the *zmspl10/14/26* triple mutant was completely hairless (Feng et al., 2021a). Three homologous of the ZmSPL transcription factors, ZmSPL10, ZmSPL14, and ZmSPL26, act synergistically to promote trichome fate in maize leaves, possibly by regulating the expression of *ZmWOX3A* and AUX-related genes (Kong et al., 2021).

The prickles on rose plants are a specialized form of trichome. The TTG1 transcription factor gene *RcTTG1* may be related to the development of rose bark, and the expression level of this gene significantly differs among different tissues (Feng et al., 2015).

Specialized trichomes of prickly pear (*Rosa roxburghii*) distributes on its leaves, stems, branches, sepals, pedicels and fruit, and the trichomes on the fruit are often called thorns; they affect the appearance and sensory quality of the plant (Wang et al., 2019a). The *RrGL1* gene of prickly pear can functionally restore the formation of trichomes in *Arabidopsis gl1* mutants, indicating that RrGL1 is involved in the development of prickly pear spines. Yeast hybridization assays indicate that RrGL1 may play a functional role in trichomes by forming an MYB-bHLH-WD40 complex. Thus, the formation of prickly pear spines is similar to that of *Arabidopsis* trichomes (Huang et al., 2019b). Moreover, there were significant differences in the expression levels of *RrGL2* in stems and fruits of different stages, indicating that this gene may be closely related to fruit spine formation and development.

Overexpression of the MYB-like gene *AmMIXTA* from snapdragon (*Antirrhinum majus*) in tobacco resulted in more trichomes, suggesting that *AmMIXTA* regulates trichome development in snapdragon (Payne et al., 2000). Under the action of the CaMV35S promoter, there were more trichomes on snapdragon leaves, further proving that *AmMIXTA* has a role in regulating snapdragon trichome development (reviewed by Martin et al., 2002). The conserved domain of the snapdragon AmMYBML1 transcription factor is almost identical to that of the AmMIXTA transcription factor, and when overexpressed in tobacco, *AmMYBML1* can promote trichome development in floral tissues (Glover et al., 2004).

## Conclusion and Outlook

Trichomes are small structures on plant arial parts. Their developmental regulation involves many hormones, transcription factors and metabolic pathways. The key genes in development of different trichome types are summarized in Supplementary Table 1. Although much research has been conducted on the fate determination, initiation, branching, elongation and maturation of trichomes in different species, there are still many knowledge gaps.

Many trichome development studies have focused on model plants such as *Arabidopsis*. Development of special forms of trichomes has been studies less, such as thorns on cucumber fruit and roses, which affect field management and picking efficiency. In addition, little research progress has been made on trichomes of monocotyledonous plants such as rice. Extensive research is needed on trichome development in various horticultural plants from multiple perspectives. Future research should focus on determining internal connections and interactions of trichomes with seeds and other organs; key regulatory factors related to trichome development; and how these regulatory factors change with environmental or developmental changes. This will provide new insights and genetic resources for plant development and crop improvement. Depending on the trichome-specific genes, corresponding promoters, such as FBP7 and E6, can also improve crop yield and quality. Therefore, the identification and application of tissue-specific promoters and specific genes are of great significance to the research of plant development and the advancement of crop genetics.

Many positive regulators and pathways in trichome development have been identified, but our understanding of negative regulatory mechanisms and key factors is still limited. For example, many C2H2 zinc finger protein transcription factors that positively regulate trichome development have been found in different species, but similar negative regulator have not been reported.

Plant hormones play an important role in trichome development. However, the regulatory mechanism of these plant hormones requires further research. AUX, BR, GA, and JA positively regulate cotton fiber initiation, whereas ABA and CTK negatively regulate cotton fiber initiation. The important roles of plant hormones in trichome development are well known, but the interactions among these hormones and their molecular mechanism requires further study. This will help identify and analyze the positive and negative factors in trichome development.

Glandular hairs development is closely related to their density on the plant surface and the types and contents of secondary metabolites. Studies in tomato, *A. annua* and cucumber have shown that C2H2 zinc finger proteins, and HD-ZIP- and MYB-type transcription factors play a key role in the glandular hair initiation and development. The biosynthesis of cytoskeleton and cutin also has an important role in the morphogenesis of glandular hairs. Interestingly, the reported glandular hair development genes often also affect the development of non-glandular hairs in plants. Therefore, it is necessary to further explore the relationship between glandular and non-glandular hair development and genes that independently regulate glandular hair development.

**FIGURE 5:**
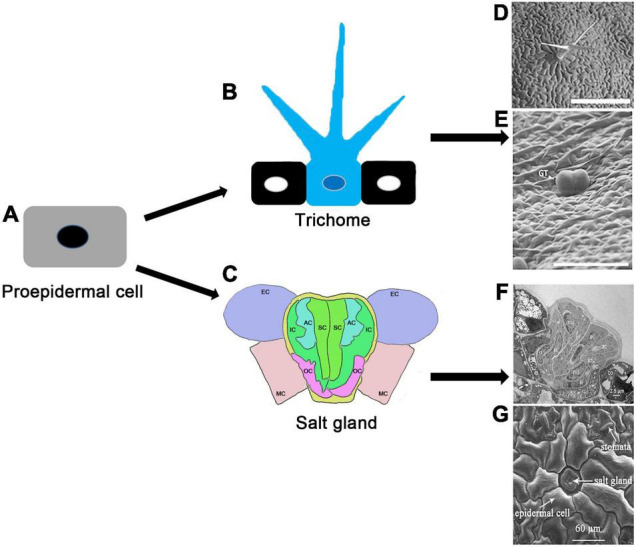
Possible evolutionary patterns in trichome and salt gland development (Breuer et al., 2009; Yuan et al., 2015b,2016a; Chalvin et al., 2020). **(A)** Schematic diagram of plant proepidermal cells; **(B)** schematic diagram of plant trichome; **(C)** schematic diagram of multicellular salt gland in *Limonium bicolor*. SC, secretory cell; AC, accessory cell; IC, inner cup cell; OC, outer cup cell; MC, mesophyll cell; EC, epidermal cell; **(D)** scanning electron microscope observation of *Arabidopsis* trichomes, 10-day-old wild-type [Columbia (Col)] on the first true leaf; **(E)** the multicellular glandular trichomes of *A. annua* leaf (adverse) were observed by scanning electron microscope. GT, glandular trichome. Scale bar = 100 μm. **(F)** Longitudinal section of salt glands in *Limonium bicolor*. Transmission electron microscope (TEM) image of salt glands of *Limonium bicolor* leaves prepared by high-pressure freezing (HPF) followed by freeze substitution (FS) and then embedded, sectioned, and stained. Scale bar = 25 μm; **(G)** morphologies of salt glands in *Limonium bicolor* leaves using environmental scanning electron microscopy (ESEM). Scale bar = 60 μm.

Similarities between trichomes and salt glands are also worthy of future study. Most plants have trichomes; however, the aerial part of the salt-secreting halophyte *Limonium bicolor* has no trichomes but has multicellular salt glands. The salt-secreting capacity of the salt glands significantly improves the salt tolerance of *L. bicolor*. The excess salt in the plant tissue can be excreted through salt glands to regulate the ion balance in the plant. In the epidermal structure of non-halophytes, such as *Arabidopsis*, tobacco and tomato, there are trichomes but not salt glands, while the leaf epidermal structure of *L. bicolor* has salt glands but not trichomes. The distribution of salt glands on *L. bicolor* leaves is similar to that of trichomes in *Arabidopsis*, they are both arranged in an interval pattern (Mishra et al., 2021). Both salt glands and trichomes differentiate earlier than stomata (Yuan et al., 2016b). In summary, salt glands and trichomes have similar temporal and spatial characteristics. In addition, transcriptome analysis of *L. bicolor* salt glands at different developmental stages showed that many differentially expressed genes in the early stage of salt gland development were homologous to the key genes in trichomes development in plants such as *Arabidopsis* (Yuan et al., 2015c). Furthermore, as an accessory structure of the plant epidermis with a secretory function, the trichomes of *A. annua* and tobacco have a strong secretory ability. Similarly, *L. bicolor* secretes salt through salt glands. Therefore, we speculate that there are certain similarities between salt-secreting glands and secretory trichomes. This raises many questions. Is there a homologous relationship between the development of trichomes and salt glands? Are the key genes that determine salt gland development homologous with the key genes in trichome development? What is the molecular evolutionary relationship between trichomes and salt glands? We propose the “common origin hypothesis” of salt glands and plant trichomes in non-halophytes, that is, there is a “common ancestor gene” that controls the differentiation of proepidermal cells, and separation may occur at a certain node in the long-term evolution of plants. One gene type controls the differentiation and development of proepidermal cells into salt glands to adapt to high salt environments, while another type controls the development into trichomes to survive under other biotic and abiotic stressors, such as arid and semiarid regions (Figure 5). To explore this hypothesis, we are currently researching salt gland development in our laboratory.

Innovation and application of cutting-edge science and technology and experimental methods will help future research on the molecular mechanisms of plant trichome development. These include chloroplast genetic engineering, nanoparticle bombardment transformation, the VIGS method, omics technology, single-cell sequencing and CRISPR-Cas9. With the rapid development and effective application of modern technologies in model plants, horticultural plants and crops, more genes and signaling pathways involved in trichome development will be identified.

## Author Contributions

GH and YL wrote this manuscript. ZY, CW, and YZ participated in the writing and modification of this manuscript. BW and GH conceptualized the idea. All authors read and approved the final manuscript.

## Conflict of Interest

The authors declare that the research was conducted in the absence of any commercial or financial relationships that could be construed as a potential conflict of interest.

## Publisher’s Note

All claims expressed in this article are solely those of the authors and do not necessarily represent those of their affiliated organizations, or those of the publisher, the editors and the reviewers. Any product that may be evaluated in this article, or claim that may be made by its manufacturer, is not guaranteed or endorsed by the publisher.

## Abbreviations

CGT: capitate glandular trichome
bHLH: basic helix-loop-helix
GA: gibberellin
CTK: cytokinin
DNA: deoxyribonucleic acid
JA: jasmonic acid
SA: salicylic acid
RNA: ribonucleic acid
mRNA: message RNA
m6A: *N6*-methyladenosine
DPA: days after anthesis
ta-siRNAs: *trans*-siRNAs
siRNA: small interfering RNA
miRNA: microRNA
ROS: reactive oxygen species
ETH: ethyne
AUX: auxin
IAA: indoleacetic acid
H_2_O_2_: hydrogen peroxide
QTL: quantitative trait locus
VIGS: virus-induced gene silencing
BR: brassinolide
RNAi: RNA interference
ABA: abscisic acid
SCW: secondary cell wall
SEM: scanning electron microscopy
Wty: warty
nWty: non-warty
6-BA: 6-benzylaminopurine
GA3: gibberellin A3
LGT: long-stalked glandular trichomes
SGT: short-stalked glandular trichomes
NGT: non-glandular trichomes
ChIP: chromatin immunoprecipitation assay
GST: glandular secretory trichome
TST: T-shaped trichome
TNGT: T-type non-glandular trichome.
