# Supplementary material for: Molecular Mechanisms of Plant Trichome Development
Source: Front Plant Sci. 2022 Jun 1;13:910228. doi: 10.3389/fpls.2022.910228 (PMC9198495; doi:10.3389/fpls.2022.910228)
Supplement: Supplementary file 1 [file Table_1.docx]

**Supplementary Table 1** Key genes for different types of trichome development

| **Gene name** | **Function** | **Species** | **References** |
| --- | --- | --- | --- |
| *GIS* | Positive regulation of trichome fate determination and initiation; Negatively regulate the branching of trichome | *Arabidopsis thaliana* | (Guan et al., 2007) (Yan et al., 2014) |
| *GIS2* | Positive regulation of trichome fate determination and initiation; Positively regulates trichome elongation and maturation | *Arabidopsis thaliana* | (Sun et al., 2015a) |
| *GIS3* | Positive regulation of trichome fate determination and initiation | *Arabidopsis thaliana* | (Sun et al., 2015a) |
| *ZFP1* | Positive regulation of trichome fate determination and initiation | *Arabidopsis thaliana* | (Sun et al., 2015a;Xie et al., 2019;Zhang et al., 2020) |
| *ZFP5* | Positive regulation of trichome fate determination and initiation | *Arabidopsis thaliana* | (Zhou et al., 2011;Zhou et al., 2012) |
| *ZFP6* | Positive regulation of trichome fate determination and initiation | *Arabidopsis thaliana* | (Zhou et al., 2013) |
| *ZFP8* | Positive regulation of trichome fate determination and initiation; Positively regulates trichome elongation and maturation | *Arabidopsis thaliana* | (Zhou et al., 2011;Zhou et al., 2012) |
| *GL1* | Positive regulation of trichome fate determination and initiation | *Arabidopsis thaliana* | (Ram et al., 2015) |
| *GL3* | Positive regulation of trichome fate determination and initiation; Negatively regulate the branching of trichome; Positively regulates trichome elongation and maturation | *Arabidopsis thaliana* | (Hung et al., 2020) |
| *EGL3* | Positive regulation of trichome fate determination and initiation; Positively regulates trichome elongation and maturation | *Arabidopsis thaliana* | (Hung et al., 2020) |
| *TTG1* | Positive regulation of trichome fate determination and initiation; Positively regulates trichome elongation and maturation | *Arabidopsis thaliana* | (Jose et al., 2018;Hung et al., 2020) |
| *SAD2* | Positive regulation of trichome fate determination and initiation | *Arabidopsis thaliana* | (Du et al., 2018) |
| *GL2* | Positive regulation of trichome fate determination and initiation | *Arabidopsis thaliana* | (Gao et al., 2008) |
| *TTG2* | Positive regulation of trichome fate determination and initiation | *Arabidopsis thaliana* | (Ishida et al., 2007) |
| *SPY* | Negative regulation of trichome fate determination and initiation; Negatively regulates trichome elongation and maturation | *Arabidopsis thaliana* | (Guan et al., 2007) |
| *GAI* | Negative regulation of trichome fate determination and initiation | *Arabidopsis thaliana* | (Guan et al., 2007) |
| *TRY* | Negative regulation of trichome fate determination and initiation | *Arabidopsis thaliana* | (Champagne and Boutry, 2017) |
| *CPC* | Negative regulation of trichome fate determination and initiation | *Arabidopsis thaliana* | (Champagne and Boutry, 2017) |
| *ETC1* | Negative regulation of trichome fate determination and initiation;Negatively regulate the branching of trichome | *Arabidopsis thaliana* | (Victor et al., 2004) |
| *SPL* | Negative regulation of trichome fate determination and initiation | *Arabidopsis thaliana* | (Yu et al., 2010;Wei et al., 2012) |
| *MYC1* | Positive regulation of trichome fate determination and initiation | *Arabidopsis thaliana* | (Pesch et al., 2013) |
| *SIM* | Negatively regulate the branching of trichome | *Arabidopsis thaliana* | (Wang et al., 2021c). |
| *CPR5* | Negatively regulate the branching of trichome | *Arabidopsis thaliana* | (Wang et al., 2021c). |
| *RBR* | Negatively regulate the branching of trichome | *Arabidopsis thaliana* | (Wang et al., 2021c). |
| *CCS52A1* | Negatively regulate the branching of trichome | *Arabidopsis thaliana* | (Remmy et al., 2010) |
| *AtMYB106* | Negatively regulate the branching of trichome | *Arabidopsis thaliana* | (Jakoby et al., 2008;Tian and Zhang, 2021) |
| *KAK* | Negatively regulate the branching of trichome | *Arabidopsis thaliana* | (El Refy et al., 2003;Schellmann and Hülskamp, 2005) |
| *CDKB1;1* | Positively regulate the branching of trichome | *Arabidopsis thaliana* | (Jiang et al., 2022) |
| *CPL3* | Positively regulate the branching of trichome | *Arabidopsis thaliana* | (Tominaga-Wada and Nukumizu, 2012) |
| *TCP14* | Positively regulate the branching of trichome | *Arabidopsis thaliana* | (Vadde et al., 2018) |
| *AtTCP15* | Positively regulate the branching of trichome | *Arabidopsis thaliana* | (Li et al., 2012) |
| *CYCA2;3* | Positively regulate the branching of trichome | *Arabidopsis thaliana* | (Li et al., 2012) |
| *ZWI* | Positively regulate the branching of trichome | *Arabidopsis thaliana* | (Chen et al., 2016b) |
| *STI* | Positively regulate the branching of trichome | *Arabidopsis thaliana* | (Victor et al., 2002;Ilgenfritz et al., 2003) |
| *TFCA/C* | Positively regulate the branching of trichome | *Arabidopsis thaliana* | (Victor et al., 2002;Ilgenfritz et al., 2003) |
| *TON2* | Positively regulate the branching of trichome | *Arabidopsis thaliana* | (Traas et al., 1995;Qiu et al., 2002;Deeks and Hussey, 2003;Ilgenfritz et al., 2003) |
| *SPIKE* | Positively regulate the branching of trichome | *Arabidopsis thaliana* | (Traas et al., 1995;Qiu et al., 2002;Deeks and Hussey, 2003;Ilgenfritz et al., 2003) |
| *ICK1*/*KRP1* | Positively regulate the branching of trichome | *Arabidopsis thaliana* | (Schnittger et al., 2003;Wang et al., 2021c) |
| *HYP6* | Positively regulate the branching of trichome | *Arabidopsis thaliana* | (El Refy et al., 2003;Schellmann and Hülskamp, 2005) |
| *RHL2* | Positively regulate the branching of trichome | *Arabidopsis thaliana* | (El Refy et al., 2003;Schellmann and Hülskamp, 2005) |
| *BRK1* | Positively regulates trichome elongation and maturation | *Arabidopsis thaliana* | (Hulskamp et al., 1994;Yang, 2002) |
| *DIS* | Positively regulates trichome elongation and maturation | *Arabidopsis thaliana* | (Hulskamp et al., 1994;Yang, 2002) |
| *TEMs* | Negatively regulates trichome elongation and maturation | *Arabidopsis thaliana* | (Fiehn et al., 2000). |
| *MYB5* | Positively regulates trichome elongation and maturation | *Arabidopsis thaliana* | (Li et al., 2009) |
| *NRX2* | Positively regulates trichome elongation and maturation | *Arabidopsis thaliana* | (FuYu et al., 2020) |
| *JMJ29* | Positively regulates trichome elongation and maturation | *Arabidopsis thaliana* | (FuYu et al., 2020) |
| *JAZ1* | Negatively regulates trichome elongation and maturation | *Arabidopsis thaliana* | (FuYu et al., 2020) |
| *GhMYB2* | Positive regulation of trichome initiation | *Gossypium hirsutum* Linn. | (Wang et al., 2004;Yong-Mei and Yu-Xian, 2011;Andreas et al., 2014;Zhao et al., 2020) |
| *GhMYB3* | Positive regulation of trichome initiation | *Gossypium hirsutum* Linn. | (Shangguan et al., 2021) |
| *GhMYB25* | Positive regulation of trichome initiation | *Gossypium hirsutum* Linn. | (Wang et al., 2021a) |
| *GhMYB25*-like | Positive regulation of trichome initiation | *Gossypium hirsutum* Linn. | (Wang et al., 2021a) |
| *GhMYB109* | Positive regulation of trichome initiation | *Gossypium hirsutum* Linn. | (Jinfeng et al., 2003) |
| *GhACO1* | Positive regulation of trichome initiation | *Gossypium hirsutum* Linn. | (Suo et al., 2003) |
| *GhACO2* | Positive regulation of trichome initiation | *Gossypium hirsutum* Linn. | (Suo et al., 2003) |
| *GhTUB1* | Positive regulation of trichome initiation and elongation | *Gossypium hirsutum* Linn. | (Suo et al., 2003) |
| *GhACT1* | Positive regulation of trichome initiation | *Gossypium hirsutum* Linn. | (Suo et al., 2003) |
| *GhCPC* | Negatively regulates trichome initiation | *Gossypium hirsutum* Linn. | (Prakash et al., 2020) |
| *GhMYC1* | Positive regulation of trichome initiation | *Gossypium hirsutum* Linn. | (Liu et al., 2015;Zhang et al., 2021a) |
| *GhTTG1/4* | Positive regulation of trichome initiation | *Gossypium hirsutum* Linn. | (Liu et al., 2015;Zhang et al., 2021a) |
| *GhHOX3* | Positive regulation of trichome initiation and elongation; Positive regulation of SCW deposition | *Gossypium hirsutum* Linn. | (Deng et al., 2012a) |
| *GhRDL1* | Positive regulation of trichome initiation and elongation | *Gossypium hirsutum* Linn. | (Deng et al., 2012a) |
| *GbRDL1* | Positive regulation of trichome initiation and elongation | *Gossypium barbadense* Linn. | (Zhang et al., 2010) |
| *GbPDF1* | Positive regulation of trichome initiation and elongation | *Gossypium barbadense* Linn. | (Jiang et al., 2012;Wang et al., 2019c) |
| *GbML1* | Positive regulation of trichome initiation | *Gossypium barbadense* Linn. | (Zhang et al., 2010) |
| *GhHOX1* | Positive regulation of trichome initiation | *Gossypium hirsutum* Linn. | (Hu et al., 2018) |
| *GaHOX1* | Positive regulation of trichome initiation | *Gossypium arboretum* Linn. | (Humphries et al., 2005;Guan et al., 2008;Zhang et al., 2010) |
| *GhHD1* | Positive regulation of trichome initiation and elongation | *Gossypium hirsutum* Linn. | (Walford et al., 2012) |
| *GhDEL65* | Positive regulation of trichome initiation | *Gossypium hirsutum* Linn. | (Shangguan et al., 2016;Sun et al., 2020) |
| *GhWRKY16* | Positive regulation of trichome initiation and elongation | *Gossypium hirsutum* Linn. | (Wang et al., 2021b) |
| *GhVIN1* | Positive regulation of trichome initiation | *Gossypium hirsutum* Linn. | (Wang et al., 2014) |
| *GhPIN3* | Positive regulation of trichome initiation | *Gossypium hirsutum* Linn. | (Li et al., 2007;Zhang et al., 2017) |
| *GhARF2b* | Positive regulation of trichome initiation and elongation | *Gossypium hirsutum* Linn. | (Liu et al., 2020a;He et al., 2021) |
| *GhARF2-1* | Positive regulation of trichome initiation | *Gossypium hirsutum* Linn. | (Xiao et al., 2018) |
| *GhARF18-1* | Positive regulation of trichome initiation | *Gossypium hirsutum* Linn. | (Xiao et al., 2018) |
| *GhJAZ2* | Negatively regulates trichome initiation | *Gossypium hirsutum* Linn. | (Xueying et al., 2014) |
| *GhGL1* | Positive regulation of trichome initiation | *Gossypium hirsutum* Linn. | (Xueying et al., 2014) |
| *GhMYC2* | Positive regulation of trichome initiation | *Gossypium hirsutum* Linn. | (Xueying et al., 2014) |
| *GhWD40* | Positive regulation of trichome initiation | *Gossypium hirsutum* Linn. | (Xueying et al., 2014) |
| *GhJI1* | Positive regulation of trichome initiation | *Gossypium hirsutum* Linn. | (Xueying et al., 2014) |
| *Gh14-3-3* | Positive regulation of trichome initiation and elongation | *Gossypium hirsutum* Linn. | (Li et al., 2013) |
| *GhBZR1* | Positive regulation of trichome initiation and elongation | *Gossypium hirsutum* Linn. | (Li et al., 2013) |
| *GhMYB212* | Positive regulation of trichome elongation | *Gossypium hirsutum* Linn. | (Sun et al., 2019) |
| *GhSWEET12* | Positive regulation of trichome elongation | *Gossypium hirsutum* Linn. | (Sun et al., 2019) |
| *GhEXPA1* | Positive regulation of trichome elongation | *Gossypium hirsutum* Linn. | (Shan et al., 2014;Shangguan et al., 2021) |
| *GhFP1* | Positive regulation of trichome elongation | *Gossypium hirsutum* Linn. | (Liu et al., 2020b) |
| *GhbHLH13* | Positive regulation of trichome elongation | *Gossypium hirsutum* Linn. | (Sun et al., 2020) |
| *GhPEL76* | Positive regulation of trichome elongation | *Gossypium hirsutum* Linn. | (Sun et al., 2020) |
| *GhPIS* | Positive regulation of trichome elongation | *Gossypium hirsutum* Linn. | (Long et al., 2018) |
| *GhAGP2* | Positive regulation of trichome elongation | *Gossypium hirsutum* Linn. | (Liu et al., 2008) |
| *GhAGP3* | Positive regulation of trichome elongation | *Gossypium hirsutum* Linn. | (Liu et al., 2008) |
| *GhAGP4* | Positive regulation of trichome elongation | *Gossypium hirsutum* Linn. | (Liu et al., 2008) |
| *GhFLA1* | Positive regulation of trichome elongation | *Gossypium hirsutum* Linn. | (Liu et al., 2008) |
| *GhMAT4* | Positive regulation of trichome elongation | *Gossypium hirsutum* Linn. | (Yu et al., 2021) |
| *GhDET2* | Positive regulation of trichome elongation | *Gossypium hirsutum* Linn. | (Luo et al., 2007) |
| *GhPAG1* | Negative regulation of trichome elongation | *Gossypium hirsutum* Linn. | (Yang et al., 2014b;Li et al., 2021) |
| *GhSLR1* | Negative regulation of trichome elongation | *Gossypium hirsutum* Linn. | (Xia et al., 2018) |
| *GhMADS11* | Positive regulation of trichome elongation | *Gossypium hirsutum* Linn. | (Wang et al., 2019c) |
| *GhMAD14* | Negative regulation of trichome elongation | *Gossypium hirsutum* Linn. | (Zhou et al., 2014) |
| *GhFSN1* | Positive regulation of SCW deposition | *Gossypium hirsutum* Linn. | (Jie et al., 2018;Zhang et al., 2018) |
| *GhIRX12* | Positive regulation of SCW deposition | *Gossypium hirsutum* Linn*.* | (Jie et al., 2018;Zhang et al., 2018) |
| *GhMYB1* | Positive regulation of SCW deposition | *Gossypium hirsutum* Linn. | (Jie et al., 2018;Zhang et al., 2018) |
| *GhGUT1* | Positive regulation of SCW deposition | *Gossypium hirsutum* Linn. | (Jie et al., 2018;Zhang et al., 2018) |
| *GhDUF231L1* | Positive regulation of SCW deposition | *Gossypium hirsutum* Linn. | (Jie et al., 2018;Zhang et al., 2018) |
| *GhMYB46_D13* | Positive regulation of SCW deposition | *Gossypium hirsutum* Linn. | (Huang et al., 2019) |
| *GhMYB46_D9* | Positive regulation of SCW deposition | *Gossypium hirsutum* Linn. | (Huang et al., 2019) |
| *GhMYB7* | Positive regulation of SCW deposition | *Gossypium hirsutum* Linn. | (Huang et al., 2016;Huang et al., 2021b) |
| *GhMYBL1* | Positive regulation of SCW deposition | *Gossypium hirsutum* Linn. | (Sun et al., 2015b) |
| *GhCesA4* | Positive regulation of SCW deposition | *Gossypium hirsutum* Linn*.* | (Kim et al., 2011;Xu et al., 2021) |
| *GhCesA7* | Positive regulation of SCW deposition | *Gossypium hirsutum* Linn. | (Yuan et al., 2015;Wang et al., 2019a;Zhang et al., 2021b) |
| *GhCesA8* | Positive regulation of SCW deposition | *Gossypium hirsutum* Linn. | (Yuan et al., 2015;Wang et al., 2019a;Zhang et al., 2021b) |
| *GhGDSL* | Positive regulation of SCW deposition | *Gossypium hirsutum* Linn. | (Yadav et al., 2017) |
| *GhMYB1* | Positive regulation of SCW deposition | *Gossypium hirsutum* Linn. | (Yadav et al., 2017) |
| *GhTCP4* | Positive regulation of SCW deposition | *Gossypium hirsutum* Linn. | (Cao et al., 2020;Huang et al., 2021a) |
| *GhHUB2* | Positive regulation of SCW deposition | *Gossypium hirsutum* Linn. | (Feng et al., 2018;Hao et al., 2018) |
| *GhKNL1* | Positive regulation of SCW deposition | *Gossypium hirsutum* Linn. | (Feng et al., 2018;Hao et al., 2018) |
| *GhBRI1* | Positive regulation of SCW deposition and cotton fiber maturation | *Gossypium hirsutum* Linn. | (Li et al., 2011;Sun et al., 2015c) |
| *GhSusA1* | Positive regulation of cotton fiber maturation | *Gossypium hirsutum* Linn. | (Bai et al., 2014;Wang et al., 2020) |
| *GLR1/OsWOX3B/DEP/NUDA /GL-1* | Positive regulation of trichome initiation | *Oryza sativa* | Shang et al., 2020b; Li et al., 2021b |
| *GLR2* | Positive regulation of trichome formation | *Oryza sativa* | (Wang et al., 2013) |
| *OsSPL10* | Positive regulation of trichome initiation | *Oryza sativa* | (Tao et al., 2019) |
| *OsTCL1* | Positive regulation of trichome initiation | *Oryza sativa* | (Kaijie et al., 2016) |
| *OsHL6* | Positive regulation of trichome initiation and elongation | *Oryza sativa* | (Sun et al., 2017) |
| *OsGL6* | Positive regulation of trichome initiation | *Oryza sativa* | (Xie et al., 2020c) |
| *OsYUCCA5* | Positive regulation of trichome initiation | *Oryza sativa* | (Xie et al., 2020c) |
| *CsGL1* | Positive regulation of trichome development (glandular and non-glandular) | *Cucumis sativus* | (Pan et al., 2015) |
| *CsTBH* | Positive regulation of trichome development (glandular and non-glandular) | *Cucumis sativus* | (Chen et al., 2014;Xue et al., 2019) |
| *CsMICT* | Positive regulation of trichome development (glandular and non-glandular) | *Cucumis sativus* | (Zhao et al., 2015;Pan et al., 2021) |
| *CsMYB6* | Negatively regulates trichome initiation (glandular and non-glandular) | *Cucumis sativus* | (Li et al., 2015) |
| *CsACS* | Positive regulation of trichome development (glandular and non-glandular) | *Cucumis sativus* | (Zhang et al., 2021c) |
| *CsTRIL* | Positively regulates the fate determination and initiation of trichome | *Cucumis sativus* | (Pan et al., 2015;Wang et al., 2016) |
| *CsGL3* | Positively regulates the fate determination and initiation of trichome (glandular and non-glandular) | *Cucumis sativus* | (Pan et al., 2015;Wang et al., 2016) |
| *CsWIN1* | Positive regulation of trichome development (glandular and non-glandular) | *Cucumis sativus* | (Pan et al., 2015;Shvachko et al., 2020) |
| *CsGL2* | Positive regulation of trichome development (glandular and non-glandular) | *Cucumis sativus* | (Pan et al., 2015;Shvachko et al., 2020) |
| *CsTRY* | Negatively regulates trichome initiation (glandular and non-glandular) | *Cucumis sativus* | (Yang et al., 2018;Yang et al., 2021) |
| *Tu* | Positive regulation of cucumber nodule formation | *Cucumis sativus* | (Yang et al., 2014a) |
| *Csa5M644580* | Positive regulation of cucumber nodule formation | *Cucumis sativus* | (Yang et al., 2014a) |
| *Csa5M224130* | Positive regulation of cucumber nodule formation | *Cucumis sativus* | (Yang et al., 2014a) |
| *CsTTG1* | Positive regulation of trichome development (glandular and non-glandular) | *Cucumis sativus* | (Chen et al., 2016a) |
| *CsHEC2* | Positively regulates the fate determination and initiation of trichome (glandular and non-glandular) | *Cucumis sativus* | (Wang et al., 2021d) |
| *CsGA20ox1* | Negative regulation of trichome elongation (glandular and non-glandular) | *Cucumis sativus* | (Li et al., 2015;Sun et al., 2018) |
| *NS* | Negative regulation of trichome development (glandular and non-glandular) | *Cucumis sativus* | (Du et al., 2020) |
| *NtZFP8* | Positive regulation of trichome development | *Nicotiana tabacum* L. | (Liu et al., 2017) |
| *NbGIS* | Positive regulation of trichome development (glandular hairs) | *Nicotiana benthamiana* L. | (Liu et al., 2018) |
| *NbMYB123-like* | Positive regulation of trichome initiation (glandular hairs) | *Nicotiana benthamiana* L. | (Liu et al., 2018) |
| *NbCycB2* | Negative regulation of trichome development | *Nicotiana benthamiana* L. | (Yang et al., 2015) |
| *NbWoolly* (*NbWo*) | Positive regulation of trichome development | *Nicotiana benthamiana* L. | (Yang et al., 2015) |
| *NbWoV* | Positive regulation of trichome development | *Nicotiana benthamiana* L. | (Wu et al., 2020) |
| *NbWo66* | Positive regulation of trichome development | *Nicotiana benthamiana* L. | (Wu et al., 2020) |
| *NtCycB2* | Negative regulation of trichome development (glandular hairs) | *Nicotiana tabacum* L. | (Wang et al., 2021e) |
| *NtJAZ* | Positive regulation of trichome initiation (glandular hairs) | *Nicotiana tabacum* L. | (Zhang et al., 2019) |
| *SlMIXTA1* | Positive regulation of trichome initiation | *Solanum Lycopersicum* | (Ewas et al., 2017;Galdon-Armero et al., 2020) |
| *SlMIXTA-like* | Positive regulation of trichome initiation | *Solanum Lycopersicum* | (Ewas et al., 2017;Galdon-Armero et al., 2020) |
| SlWOOLLY (*SlWo*) | Positive regulation of trichome development (glandular hairs) | *Solanum Lycopersicum* | (Yanna et al., 2015) |
| *SlCycB2* | Positive regulation of trichome development (glandular hairs) | *Solanum Lycopersicum* | (Yanna et al., 2015) |
| *SlCD2* | Positive regulation of trichome development (glandular hairs) | *Solanum Lycopersicum* | (Nadakuduti et al., 2012) |
| *HDZIPIV8* | Positive regulation of trichome development (glandular and non-glandular) | *Solanum Lycopersicum* | (Xie et al., 2020b) |
| *Hairless-2* (*hl-2*) | Positive regulation of trichome development (glandular and non-glandular) | *Solanum Lycopersicum* | (Xie et al., 2020a) |
| *SlHZ45* | Positive regulation of trichome development (glandular hairs) | *Solanum Lycopersicum* | (Zhang et al., 2014) |
| *Hair* (*H*) | Positive regulation of trichome initiation and elongation (glandular hairs) | *Solanum Lycopersicum* | (Chang et al., 2018) |
| *SlZFP8-like* (*SlZFP8L*) | Positive regulation of trichome initiation and elongation (glandular hairs) | *Solanum Lycopersicum* | (Zheng et al., 2021) |
| *SlZFP6* | Positive regulation of trichome initiation and elongation (glandular hairs) | *Solanum Lycopersicum* | (Zheng et al., 2021) |
| *SlMYC1* | Positive regulation of trichome development (glandular hairs) | *Solanum Lycopersicum* | (Xu et al., 2019) |
| *SlJAZ2* | Negative regulation of trichome initiation (glandular hairs) | *Solanum Lycopersicum* | (Xu et al., 2019) |
| *SlbHLH95* | Negative regulation of trichome initiation (glandular hairs) | *Solanum Lycopersicum* | (Chen et al., 2020) |
| *SlCOI1* | Positive regulation of trichome development (glandular hairs) | *Solanum Lycopersicum* | (Thines et al., 2007) |
| *SlIAA15* | Positive regulation of trichome development (glandular and non-glandular) | *Solanum Lycopersicum* | (Deng et al., 2012b) |
| *SlARF3* | Positive regulation of trichome development (glandular and non-glandular) | *Solanum Lycopersicum* | (Deng et al., 2012b) |
| *SlJAZ4* | Negative regulation of trichome elongation | *Solanum Lycopersicum* | (Hua et al., 2021) |
| *SlHD8* | Positive regulation of trichome elongation | *Solanum Lycopersicum* | (Hua et al., 2021) |
| *AaMIXTA1* | Positive regulation of trichome initiation (glandular hairs) | *Artemisia annua* | (Matías‐Hernández et al., 2017) |
| *AaMYB1* | Positive regulation of trichome development (glandular hairs) | *Artemisia annua* | (Matías‐Hernández et al., 2017) |
| *AaMYB16* | Positive regulation of trichome initiation (glandular hairs) | *Artemisia annua* | (Xie et al., 2021a) |
| *AaMYB5* | Negative regulation of trichome initiation (glandular hairs) | *Artemisia annua* | (Xie et al., 2021a) |
| *AaHD1* | Positive regulation of trichome initiation (glandular hairs) | *Artemisia annua* | (Xie et al., 2021a) |
| *AaHD8* | Positive regulation of trichome initiation (glandular hairs) | *Artemisia annua* | (Yan et al., 2018) |
| *AaGSW2* | Positive regulation of trichome initiation (glandular hairs) | *Artemisia annua* | (Xie et al., 2021b) |
| *TAR1* | Negative regulation of trichome development (glandular hairs) | *Artemisia annua* | (Tan et al., 2015) |
| *BGL1* | Positive regulation of trichome development (glandular and non-glandular) | *Artemisia annua* | (Singh et al., 2016) |
| *AaJAZ8* | Negative regulation of trichome development (glandular hairs) | *Artemisia annua* | (Yan et al., 2017) |
| *AaSAP1* | Positive regulation of trichome development (glandular hairs) | *Artemisia annua* | (Wang et al., 2019b) |
| *TLR1* | Negative regulation of trichome development (glandular hairs) | *Artemisia annua* | (Lv et al., 2022) |
| *TLR2* | Negative regulation of trichome development (glandular hairs) | *Artemisia annua* | (Lv et al., 2022) |
| *AaTAR2* | Positive regulation of trichome development (glandular hairs) | *Artemisia annua* | (Zhou et al., 2020) |
| *AaSPL9* | Positive regulation of trichome development (glandular hairs) | *Artemisia annua* | (He et al., 2022) |

**References**

Andreas, G., Kazuko, Y., T, T.L., and Peter, C.C. (2014). Characterization of an apple TT2-type R2R3 MYB transcription factor functionally similar to the poplar proanthocyanidin regulator PtMYB134. *Planta* 240**,** 497-511.

Bai, W.Q., Xiao, Y.H., Zhao, J., Song, S.Q., Hu, L., Zeng, J.Y., Li, X.B., Hou, L., Luo, M., Li, D.M., and Pei, Y. (2014). Gibberellin overproduction promotes sucrose synthase expression and secondary cell wall deposition in cotton fibers. *PLoS One* 9**,** e96537.

Cao, J.-F., Zhao, B., Huang, C.-C., Chen, Z.-W., Zhao, T., Liu, H.-R., Hu, G.-J., Shangguan, X.-X., Shan, C.-M., and Wang, L.-J. (2020). The miR319-targeted GhTCP4 promotes the transition from cell elongation to wall thickening in cotton fiber. *Molecular Plant* 13**,** 1063-1077.

Champagne, A., and Boutry, M. (2017). A comprehensive proteome map of glandular trichomes of hop (Humulus lupulus L.) female cones: Identification of biosynthetic pathways of the major terpenoid-related compounds and possible transport proteins. *Proteomics* 17**,** 8.

Chang, J., Yu, T., Yang, Q., Li, C., Xiong, C., Gao, S., Xie, Q., Zheng, F., Li, H., Tian, Z., Yang, C., and Ye, Z. (2018). Hair, encoding a single C2H2 zinc-finger protein, regulates multicellular trichome formation in tomato. *Plant J* 96**,** 90-102.

Chen, C., Liu, M., Jiang, L., Liu, X., Zhao, J., Yan, S., Yang, S., Ren, H., Liu, R., and Zhang, X. (2014). Transcriptome profiling reveals roles of meristem regulators and polarity genes during fruit trichome development in cucumber (Cucumis sativus L.). *J Exp Bot* 65**,** 4943-4958.

Chen, C., Yin, S., Liu, X., Liu, B., Yang, S., Xue, S., Cai, Y., Black, K., Liu, H., and Dong, M. (2016a). The WD-repeat protein CsTTG1 regulates fruit wart formation through interaction with the homeodomain-leucine zipper I protein Mict. *Plant Physiol* 171**,** 1156-1168.

Chen, L., Peng, Y., Tian, J., Wang, X., Kong, Z., Mao, T., Yuan, M., and Li, Y. (2016b). TCS1, a Microtubule-Binding Protein, Interacts with KCBP/ZWICHEL to Regulate Trichome Cell Shape in Arabidopsis thaliana. *Plos Genetics* 12**,** e1006266.

Chen, Y., Su, D., Li, J., Ying, S., Deng, H., He, X., Zhu, Y., Li, Y., Chen, Y., Pirrello, J., Bouzayen, M., Liu, Y., and Liu, M. (2020). Overexpression of bHLH95, a basic helix-loop-helix transcription factor family member, impacts trichome formation via regulating gibberellin biosynthesis in tomato. *Journal of Experimental Botany* 71**,** 3450-3462.

Deeks, M.J., and Hussey, P.J. (2003). Arp2/3 and ‘The Shape of things to come’. *Curr Opin Plant Biol* 6**,** 561-567.

Deng, F., Tu, L., Tan, J., Li, Y., Nie, Y., and Zhang, X. (2012a). GbPDF1 is involved in cotton fiber initiation via the core cis-element HDZIP2ATATHB2. *Plant Physiol* 158**,** 890-904.

Deng, W., Yang, Y., Ren, Z., Audran‐Delalande, C., Mila, I., Wang, X., Song, H., Hu, Y., Bouzayen, M., and Li, Z. (2012b). The tomato SlIAA15 is involved in trichome formation and axillary shoot development. *New Phytol* 194**,** 379-390.

Du, H., Wang, G., Pan, J., Chen, Y., Xiao, T., Zhang, L., Zhang, K., Wen, H., Xiong, L., Yu, Y., He, H., Pan, J., Cai, R., and Lawson, T. (2020). The HD-ZIP IV transcription factor Tril regulates fruit spine density through gene dosage effects in cucumber. *Journal of Experimental Botany* 71**,** 6297-6310.

Du, X., Huang, G., He, S., Yang, Z., Sun, G., Ma, X., Li, N., Zhang, X., Sun, J., Liu, M., Jia, Y., Pan, Z., Gong, W., Liu, Z., Zhu, H., Ma, L., Liu, F., Yang, D., Wang, F., Fan, W., Gong, Q., Peng, Z., Wang, L., Wang, X., Xu, S., Shang, H., Lu, C., Zheng, H., Huang, S., Lin, T., Zhu, Y., and Li, F. (2018). Resequencing of 243 diploid cotton accessions based on an updated A genome identifies the genetic basis of key agronomic traits. *Nature Genetics* 50**,** 796-802.

El Refy, A., Perazza, D., Zekraoui, L., Valay, J.G., Bechtold, N., Brown, S., Hülskamp, M., Herzog, M., and Bonneville, J.M. (2003). The Arabidopsis KAKTUS gene encodes a HECT protein and controls the number of endoreduplication cycles. *Mol Genet Genomics* 270**,** 403-414.

Ewas, M., Gao, Y., Ali, F., Nishawy, E.M., Shahzad, R., Subthain, H., Amar, M., Martin, C., and Luo, J. (2017). RNA-seq reveals mechanisms of SlMX1 for enhanced carotenoids and terpenoids accumulation along with stress resistance in tomato. *Science Bulletin* 62**,** 476-485.

Feng, H., Li, X., Chen, H., Deng, J., Zhang, C., Liu, J., Wang, T., Zhang, X., and Dong, J. (2018). GhHUB2, a ubiquitin ligase, is involved in cotton fiber development via the ubiquitin–26S proteasome pathway. *J Exp Bot* 69**,** 5059-5075.

Fiehn, O., Kopka, J., Dörmann, P., Altmann, T., Trethewey, R.N., and Willmitzer, L. (2000). Metabolite profiling for plant functional genomics. *Nature Biotechnology: The Science and Business of Biotechnology* 18**,** 1157-1161.

Fuyu, H., Jianhao, C., Yunru, F., Youcheng, L., Songguang, Y., and Keqiang, W. (2020). Arabidopsis JMJ29 is involved in trichome development by regulating the core trichome initiation gene GLABRA3. *Plant J* 103**,** 1735-1743.

Galdon-Armero, J., Arce-Rodriguez, L., Downie, M., Li, J., and Martin, C. (2020). A Scanning Electron Micrograph-based Resource for Identification of Loci Involved in Epidermal Development in Tomato: Elucidation of a New Function for the Mixta-like Transcription Factor in Leaves. *Plant Cell* 32**,** 1414-1433.

Gao, Y., Gong, X., Cao, W., Zhao, J., Fu, L., Wang, X., Schumaker, K.S., and Guo, Y. (2008). SAD2 in Arabidopsis functions in trichome initiation through mediating GL3 function and regulating GL1, TTG1 and GL2 expression. *J Integr Plant Biol* 50**,** 906-917.

Guan, X., Yu, N., Shangguan, X., Wang, S., Lu, S., Wang, L., and Chen, X. (2007). Arabidopsis trichome research sheds light on cotton fiber development mechanisms. *Chinese Science Bulletin* 52**,** 1734-1741.

Guan, X.Y., Li, Q.J., Shan, C.M., Wang, S., Mao, Y.B., Wang, L.J., and Chen, X.Y. (2008). The HD‐Zip IV gene GaHOX1 from cotton is a functional homologue of the Arabidopsis GLABRA2. *Physiol Plant* 134**,** 174-182.

Hao, F., Xin, L., Hong, C., Jie, D., Chaojun, Z., Ji, L., Tao, W., Xueyan, Z., and Jiangli, D. (2018). GhHUB2, a ubiquitin ligase, is involved in cotton fiber development via the ubiquitin-26S proteasome pathway. *J Exp Bot* 69**,** 5059-5075.

He, P., Zhang, Y., Li, H., Fu, X., Shang, H., Zou, C., Friml, J., and Xiao, G. (2021). GhARF16-1 modulates leaf development by transcriptionally regulating theGhKNOX2-1gene in cotton. *Plant Biotechnology Journal* 19**,** 548-562.

He, Y.L., Fu, X.Q., Li, L., Sun, X.F., Tang, K.X., and Zhao, J.Y. (2022). AaSPL9 affects glandular trichomes initiation by positively regulating expression of AaHD1 in Artemisia annua L. *Plant Science* 317, 111172.

Hu, H., Wang, M., Ding, Y., Zhu, S., Zhao, G., Tu, L., and Zhang, X. (2018). Transcriptomic repertoires depict the initiation of lint and fuzz fibres in cotton ( Gossypium hirsutum L.). *Plant Biotechnology Journal* 16**,** 1002-1012.

Hua, B., Chang, J., Xu, Z., Han, X., Xu, M., Yang, M., Yang, C., Ye, Z., and Wu, S. (2021). HOMEODOMAIN PROTEIN8 mediates jasmonate-triggered trichome elongation in tomato. *New Phytol* 230**,** 1063-1077.

Huang, G., Huang, J.-Q., Chen, X.-Y., and Zhu, Y.-X. (2021a). Recent advances and future perspectives in cotton research. *Annu Rev Plant Biol* 72, 437-462.

Huang, J., Chen, F., Guo, Y., Gan, X., Yang, M., Zeng, W., Persson, S., Li, J., and Xu, W. (2021b). GhMYB7 promotes secondary wall cellulose deposition in cotton fibres by regulating GhCesA gene expression through three distinct cis‐elements. *New Phytol* 232**,**1718-1737.

Huang, J., Chen, F., Wu, S., Li, J., and Xu, W. (2016). Cotton GhMYB7 is predominantly expressed in developing fibers and regulates secondary cell wall biosynthesis in transgenic Arabidopsis. *Sci China Life Sci* 59**,** 194-205.

Huang, J., Guo, Y., Sun, Q., Zeng, W., Li, J., Li, X., and Xu, W. (2019). Genome-Wide Identification of R2R3-MYB Transcription Factors Regulating Secondary Cell Wall Thickening in Cotton Fiber Development. *Plant Cell Physiol* 60**,** 687-701.

Hulskamp, M., Misra, S., and Jurgens, G. (1994). Genetic dissection of trichome cell development in Arabidopsis. *Cell* 76**,** 555-566.

Humphries, J.A., Walker, A.R., Timmis, J.N., and Orford, S.J. (2005). Two WD-repeat genes from cotton are functional homologues of the Arabidopsis thaliana TRANSPARENT TESTA GLABRA1 ( TTG1 ) gene. *Plant Mol Biol* 57**,** 67-81.

Hung, F.Y., Chen, J.H., Feng, Y.R., Lai, Y.C., Yang, S.G., and Wu, K.Q. (2020). Arabidopsis JMJ29 is involved in trichome development by regulating the core trichome initiation geneGLABRA3. *Plant Journal* 103**,** 1735-1743.

Ilgenfritz, H., Bouyer, D., Schnittger, A., Mathur, J., Kirik, V., Schwab, B., Chua, N.-H., Jürgens, G., and Hülskamp, M. (2003). The Arabidopsis STICHEL Gene Is a Regulator of Trichome Branch Number and Encodes a Novel Protein. *Plant Physiol* 131**,** 643-655.

Ishida, T., Hattori, S., Sano, R., Inoue, K., Shirano, Y., Hayashi, H., Shibata, D., Sato, S., Kato, T., Tabata, S., Okada, K., and Wada, T. (2007). Arabidopsis TRANSPARENT TESTA GLABRA2 is directly regulated by R2R3 MYB transcription factors and is involved in regulation of GLABRA2 transcription in epidermal differentiation. *Plant Cell* 19**,** 2531-2543.

Jakoby, M.J., Falkenhan, D., Mader, M.T., Brininstool, G., Wischnitzki, E., Platz, N., Hudson, A., Hulskamp, M., Larkin, J., and Schnittger, A. (2008). Transcriptional profiling of mature Arabidopsis trichomes reveals that NOECK encodes the MIXTA-like transcriptional regulator MYB106. *Plant Physiology* 148**,** 1583-1602.

Jiang, S., Wei, J., Li, N., Wang, Z., Zhang, Y., Xu, R., Zhou, L., Huang, X., Wang, L., Guo, S., Wang, Y., Song, C.P., Qian, W., and Li, Y. (2022). The UBP14-CDKB1; 1-CDKG2 cascade controls endoreduplication and cell growth in Arabidopsis. *Plant Cell* 6**,** koac002.

Jiang, Y., Guo, W., Zhu, H., Ruan, Y.L., and Zhang, T. (2012). Overexpression of GhSusA1 increases plant biomass and improves cotton fiber yield and quality. *Plant Biotechnology Journal* 10**,** 301-312.

Jie, Z., Geng-Qing, H., Dan, Z., Jing-Qiu, Y., Yang, L., Shan, H., and Xue-Bao, L. (2018). The cotton (Gossypium hirsutum) NAC transcription factor (FSN1) as a positive regulator participates in controlling secondary cell wall biosynthesis and modification of fibers. *New Phytol* 217**,** 625-640.

Jinfeng, S., Xiaoe, L., Li, P., Yansheng, Z., and Yongbiao, X. (2003). Identification of GhMYB109 encoding a R2R3 MYB transcription factor that expressed specifically in fiber initials and elongating fibers of cotton (Gossypium hirsutum L.). *Biophysica Acta-Gene Structure and Expression* 1630**,** 25-34.

Jose, D.-V., Luis, C.-P.J., Carlos, M.-G.J., and R, A.-B.E. (2018). Modeling the Epigenetic Landscape in Plant Development. *Methods in molecular biology (Clifton, N.J.)* 1819**,** 357-383.

Kaijie, Z., Hainan, T., Qingnan, H., Hongyan, G., Li, Y., Ling, C., Xutong, W., Bao, L., and Shucai, W. (2016). Ectopic expression of R3 MYB transcription factor gene OsTCL1 in Arabidopsis, but not rice, affects trichome and root hair formation. *Scientific Reports* 6**,**19254.

Kim, H.J., Murai, N., Fang, D.D., and Triplett, B.A. (2011). Functional analysis of Gossypium hirsutum cellulose synthase catalytic subunit 4 promoter in transgenic Arabidopsis and cotton tissues. *Plant Sci* 180**,** 323-332.

Li, B., Li, D.-D., Zhang, J., Xia, H., Wang, X.-L., Li, Y., and Li, X.-B. (2013). Cotton AnnGh3 Encoding an Annexin Protein is Preferentially Expressed in Fibers and Promotes Initiation and Elongation of Leaf Trichomes in Transgenic Arabidopsis. *Journal of Integrative Plant Biology* 55**,** 902-916.

Li, H.B., Qin, Y.M., Pang, Y., Song, W.Q., Mei, W.Q., and Zhu, Y.X. (2007). A cotton ascorbate peroxidase is involved in hydrogen peroxide homeostasis during fibre cell development. *New Phytologist* 175**,** 462-471.

Li, J., Wang, X., Jiang, R., Dong, B., Fang, S., Li, Q., Lv, Z., and Chen, W. (2021). Phytohormone-Based Regulation of Trichome Development. *Front Plant Sci* 12**,** 734776.

Li, Q., Cao, C.X., Zhang, C.J., Zheng, S.S., Wang, Z.H., Wang, L.N., and Ren, Z.H. (2015). The identification of Cucumis sativus Glabrous 1 (CsGL1) required for the formation of trichomes uncovers a novel function for the homeodomain-leucine zipper I gene. *Journal of Experimental Botany* 66**,** 2515-2526.

Li, S.F., Milliken, O.N., Pham, H., Seyit, R., Napoli, R., Preston, J., Koltunow, A.M., and Parisha, R.W. (2009). The Arabidopsis MYB5 Transcription Factor Regulates Mucilage Synthesis, Seed Coat Development, and Trichome Morphogenesis. *Plant Cell* 21**,** 72-89.

Li, Y., Ning, H., Zhang, Z., Wu, Y., Jiang, J., Su, S., Tian, F., and Li, X. (2011). A cotton gene encoding novel MADS-box protein is preferentially expressed in fibers and functions in cell elongation. *Acta Biochim Biophys Sin* 43**,** 607.

Li, Z.Y., Li, B., and Dong, A.W. (2012). The Arabidopsis transcription factor AtTCP15 regulates endoreduplication by modulating expression of key cell-cycle genes. *Mol Plant* 5**,** 270-280.

Liu, B., Zhu, Y., and Zhang, T. (2015). The R3-MYB Gene GhCPC Negatively Regulates Cotton Fiber Elongation. *Plos One* 10**,** e0116272.

Liu, D., Tu, L., Li, Y., Wang, L., Zhu, L., and Zhang, X. (2008). Genes Encoding Fasciclin-Like Arabinogalactan Proteins are Specifically Expressed During Cotton Fiber Development. *Plant Molecular Biology Reporter* 26**,** 98-113.

Liu, N., Wu, S., Li, Z., Khan, A.Q., Hu, H., Zhang, X., and Tu, L. (2020a). Repression of microRNA 160 results in retarded seed integument growth and smaller final seed size in cotton. *Crop Journal* 8**,** 602-612.

Liu, Y., Liu, D., Hu, R., Hua, C., Ali, I., Zhang, A., Liu, B., Wu, M., Huang, L., and Gan, Y. (2017). AtGIS, a C2H2 zinc-finger transcription factor from Arabidopsis regulates glandular trichome development through GA signaling in tobacco. *Biochemical and Biophysical Research Communications* 483**,** 209.

Liu, Y.H., Liu, D.D., Khan, A.R., Liu, B.H., Wu, M.J., Huang, L.L., Wu, J.Y., Song, G., Ni, H.W., Ying, H.M., Yu, H., and Gan, Y.B. (2018). NbGIS regulates glandular trichome initiation through GA signaling in tobacco. *Plant Molecular Biology* 98**,** 153-167.

Liu, Z.H., Chen, Y., Wang, N.N., Chen, Y.H., Wei, N., Lu, R., Li, Y., and Li, X.B. (2020b). A basic helix–loop–helix protein (GhFP1) promotes fibre elongation of cotton (Gossypium hirsutum) by modulating brassinosteroid biosynthesis and signalling. *New Phytol* 225**,** 2439-2452.

Long, Q., Yue, F., Liu, R., Song, S., Li, X., Ding, B., Yan, X., and Pei, Y. (2018). The phosphatidylinositol synthase gene (GhPIS) contributes to longer, stronger, and finer fibers in cotton. *Molecular Genetics and Genomics* 293**,** 1139-1149.

Luo, M., Xiao, Y., Li, X., Lu, X., Deng, W., Li, D., Hou, L., Hu, M., Li, Y., and Pei, Y. (2007). GhDET2, a steroid 5α‐reductase, plays an important role in cotton fiber cell initiation and elongation. *Plant J* 51**,** 419-430.

Lv, Z., Li, J., Qiu, S., Qi, F., Su, H., Bu, Q., Jiang, R., Tang, K., Zhang, L., and Chen, W. (2022). The transcription factors TLR1 and TLR2 negatively regulate trichome density and artemisinin levels in Artemisia annua. *Journal of integrative plant biology* , doi: 10.1111/jipb.13258.

Matías‐Hernández, L., Jiang, W., Yang, K., Tang, K., Brodelius, P.E., and Pelaz, S. (2017). Aa MYB 1 and its orthologue At MYB 61 affect terpene metabolism and trichome development in Artemisia annua and Arabidopsis thaliana. *Plant J* 90**,** 520-534.

Nadakuduti, S.S., Pollard, M., Kosma, D.K., Allen, C., Jr., Ohlrogge, J.B., and Barry, C.S. (2012). Pleiotropic Phenotypes of the sticky peel Mutant Provide New Insight into the Role of CUTIN DEFICIENT2 in Epidermal Cell Function in Tomato. *Plant Physiology* 159**,** 945-960.

Pan, J., Zhang, L., Chen, G., Wen, H., Chen, Y., Du, H., Zhao, J., He, H., Lian, H., and Chen, H. (2021). Study of micro-trichome (mict) reveals novel connections between transcriptional regulation of multicellular trichome development and specific metabolism in cucumber. *Hortic Res* 8**,** 1-12.

Pan, Y.P., Bo, K.L., Cheng, Z.H., and Weng, Y.Q. (2015). The loss-of-function GLABROUS 3 mutation in cucumber is due to LTR-retrotransposon insertion in a class IV HD-ZIP transcription factor gene CsGL3 that is epistatic over CsGL1. *Bmc Plant Biology* 15**,** 302.

Pesch, M., Schultheiß, I., Digiuni, S., Uhrig, J.F., and Hülskamp, M. (2013). Mutual control of intracellular localisation of the patterning proteins AtMYC1, GL1 and TRY/CPC in Arabidopsis. *Development* 140**,** 3456-3467.

Prakash, P., Srivastava, R., Prasad, P., Tiwari, V.K., Kumar, A., Pandey, S., and Sawant, S.V. (2020). Trajectories of cotton fiber initiation: a regulatory perspective. *Preprints* doi: 10.20944/preprints202011.0060.v1.

Qiu, J.-L., Jilk, R., Marks, M.D., and Szymanski, D.B. (2002). The Arabidopsis SPIKE1 Gene Is Required for Normal Cell Shape Control and Tissue Development. *The Plant Cell* 14**,** 101–118.

Ram, B.D., Qing, W., Wolfgang, F., Bernhard, S., Sven, G., and Andreas, R. (2015). High resolution mass spectrometry imaging of plant tissues: towards a plant metabolite atlas. *The Analyst* 140**,** 7696-7696.

Remmy, K., D, W.J., Alice, S.L., Jing, Z., Lieven, D.V., and C, L.J. (2010). SIAMESE cooperates with the CDH1-like protein CCS52A1 to establish endoreplication in Arabidopsis thaliana trichomes. *Genetics* 185, 257-268.

Schellmann, S., and Hülskamp, M. (2005). Epidermal differentiation: trichomes in Arabidopsis as a model system. *Int J Dev Biol* 49**,** 579-584.

Schnittger, A., Weinl, C., Bouyer, D., Schobinger, U., and Hulskamp, M. (2003). Misexpression of the cyclin-dependent kinase inhibitor ICK1/KRP1 in single-celled Arabidopsis trichomes reduces endoreduplication and cell size and induces cell death. *Plant Cell* 15**,** 303-315.

Shan, C.-M., Shangguan, X.-X., Zhao, B., Zhang, X.-F., Chao, L.-M., Yang, C.-Q., Wang, L.-J., Zhu, H.-Y., Zeng, Y.-D., Guo, W.-Z., Zhou, B.-L., Hu, G.-J., Guan, X.-Y., Chen, Z.J., Wendel, J.F., Zhang, T.-Z., and Chen, X.-Y. (2014). Control of cotton fibre elongation by a homeodomain transcription factor GhHOX3. *Nat Commun* 5**,** 5519.

Shangguan, X., Yang, Q., Wu, X., and Cao, J. (2021). Function analysis of a cotton R2R3 MYB transcription factor GhMYB3 in regulating plant trichome development. *Plant Biol (Stuttg)* 23**,** 1118-1127.

Shangguan, X.X., Yang, C.Q., Zhang, X.F., and Wang, L.J. (2016). Functional characterization of a basic helix‐loop‐helix (bHLH) transcription factor GhDEL65 from cotton (Gossypium hirsutum). *Physiol Plant* 158**,** 200-212.

Shvachko, N., Semilet, T., and Tikhonova, N. (2020). Trichomes of Higher Plants: Homologous Series in Hereditary Variability and Molecular Genetic Mechanisms. *Physiol Plant* 56**,** 1359-1370.

Singh, N.D., Kumar, S., and Daniell, H. (2016). Expression of β-glucosidase increases trichome density and artemisinin content in transgenic Artemisia annua plants. *Plant Biotechnol J* 14**,** 1034-1045.

Sun, H., Hao, P., Gu, L., Cheng, S., Wang, H., Wu, A., Ma, L., Wei, H., and Yu, S. (2020). Pectate lyase-like Gene GhPEL76 regulates organ elongation in Arabidopsis and fiber elongation in cotton. *Plant Sci* 293**,** 110395.

Sun, H., Pang, B.Y., Yan, J., Wang, T., Wang, L.N., Chen, C.H., Li, Q., and Ren, Z.H. (2018). Comprehensive Analysis of Cucumber Gibberellin Oxidase Family Genes and Functional Characterization of CsGA20ox1 in Root Development in Arabidopsis. *International Journal of Molecular Sciences* 19**,** 3135.

Sun, L., Zhang, A., Zhou, Z., Zhao, Y., Yan, A., Bao, S., Yu, H., and Gan, Y. (2015a). GLABROUS INFLORESCENCE STEMS3 (GIS3) regulates trichome initiation and development in Arabidopsis. *New Phytologist* 206**,** 220-230.

Sun, W., Gao, D., Xiong, Y., Tang, X., Xiao, X., Wang, C., and Yu, S. (2017). Hairy Leaf 6, an AP2/ERF Transcription Factor, Interacts with OsWOX3B and Regulates Trichome Formation in Rice. *Mol Plant* 10**,** 1417-1433.

Sun, W., Gao, Z., Wang, J., Huang, Y., Chen, Y., Li, J., Lv, M., Wang, J., Luo, M., and Zuo, K. (2019). Cotton fiber elongation requires the transcription factor Gh MYB 212 to regulate sucrose transportation into expanding fibers. *New Phytol* 222**,** 864-881.

Sun, X., Gong, S.Y., Nie, X.Y., Li, Y., Li, W., Huang, G.Q., and Li, X.B. (2015b). A R2R3-MYB transcription factor that is specifically expressed in cotton (Gossypium hirsutum) fibers affects secondary cell wall biosynthesis and deposition in transgenic Arabidopsis. *Physiol Plant* 154**,** 420-432.

Sun, Y., Veerabomma, S., Fokar, M., Abidi, N., Hequet, E., Payton, P., and Allen, R.D. (2015c). Brassinosteroid signaling affects secondary cell wall deposition in cotton fibers. *Industrial Crops and Products* 65**,** 334-342.

Suo, J., Liang, X., Pu, L., Zhang, Y., and Xue, Y. (2003). Identification of GhMYB109 encoding a R2R3 MYB transcription factor that expressed specifically in fiber initials and elongating fibers of cotton (Gossypium hirsutum L.). *Biochim Biophys Acta* 1630**,** 25-34.

Tan, H., Xiao, L., Gao, S., Li, Q., Chen, J., Xiao, Y., Ji, Q., Chen, R., Chen, W., and Zhang, L. (2015). TRICHOME AND ARTEMISININ REGULATOR 1 Is Required for Trichome Development and Artemisinin Biosynthesis in Artemisia annua. *Mol Plant* 8**,** 1396-1411.

Tao, L., Yali, Z., Zilong, S., Shibo, Y., Haibing, S., Xiaoya, Z., Gege, L., and Weiren, W. (2019). OsSPL10 , a SBP-Box Gene, Plays a Dual Role in Salt Tolerance and Trichome Formation in Rice ( Oryza sativa L.). *G3 (Bethesda)* 9**,** 4107-4114.

Thines, B., Katsir, L., Melotto, M., Niu, Y., Mandaokar, A., Liu, G., Nomura, K., He, S.Y., Howe, G.A., and Browse, J. (2007). JAZ repressor proteins are targets of the SCFCOI1 complex during jasmonate signalling. *Nature* 448, 661–665.

Tian, Y., and Zhang, T. (2021). MIXTAs and phytohormones orchestrate cotton fiber development. *Curr Opin Plant Biol* 59**,** 101975.

Tominaga-Wada, R., and Nukumizu, Y. (2012). The CAPRICE-LIKE MYB gene family cooperatively controls trichome branching and clustering in Arabidopsis. *Plant Biotechnology* 29**,** 407-410.

Traas, J., Bellini, C., Nacry, P., Kronenberger, J., Bouchez, D., and Caboche, M. (1995). Normal differentiation patterns in plants lacking microtubular preprophase bands. *Nature* 375**,** 676-677.

Vadde, B.V.L., Challa, K.R., and Nath, U. (2018). The TCP 4 transcription factor regulates trichome cell differentiation by directly activating GLABROUS INFLORESCENCE STEMS in Arabidopsis thaliana. *Plant J* 93**,** 259-269.

Victor, K., E, G.P., Jaideep, M., Irene, K., Klaus, A., Nicole, B., Michel, H., Jean-Marc, B., and Martin, H. (2002). The Arabidopsis TUBULIN-FOLDING COFACTOR A gene is involved in the control of the alpha/beta-tubulin monomer balance. *Plant Cell* 14, 2265-2276.

Victor, K., Marissa, S., Martin, H., and John, S. (2004). The ENHANCER OF TRY AND CPC1 gene acts redundantly with TRIPTYCHON and CAPRICE in trichome and root hair cell patterning in Arabidopsis. *Dev Biol* 268**,** 506-13.

Walford, S.A., Wu, Y., Llewellyn, D.J., and Dennis, E.S. (2012). Epidermal cell differentiation in cotton mediated by the homeodomain leucine zipper gene, GhHD-1. *Plant J* 71**,** 464-478.

Wang, L., Cook, A., Patrick, J.W., Chen, X.Y., and Ruan, Y.L. (2014). Silencing the vacuolar invertase gene GhVIN1 blocks cotton fiber initiation from the ovule epidermis, probably by suppressing a cohort of regulatory genes via sugar signaling. *Plant J* 78**,** 686-696.

Wang, L., Kartika, D., and Ruan, Y.L. (2021a). Looking into ‘hair tonics’ for cotton fiber initiation. *New Phytol* 229**,** 1844-1851.

Wang, L., Wang, G., Long, L., Altunok, S., Feng, Z., Wang, D., Khawar, K.M., and Mujtaba, M. (2020). Understanding the role of phytohormones in cotton fiber development through omic approaches; recent advances and future directions. *Int J Biol Macromol* 163**,** 1301-1313.

Wang, M., Tu, L., Yuan, D., Zhu, D., Shen, C., Li, J., Liu, F., Pei, L., Wang, P., Zhao, G., Ye, Z., Huang, H., Yan, F., Ma, Y., Zhang, L., Liu, M., You, J., Yang, Y., Liu, Z., Huang, F., Li, B., Qiu, P., Zhang, Q., Zhu, L., Jin, S., Yang, X., Min, L., Li, G., Chen, L.L., Zheng, H., Lindsey, K., Lin, Z., Udall, J.A., and Zhang, X. (2019a). Reference genome sequences of two cultivated allotetraploid cottons, Gossypium hirsutum and Gossypium barbadense. *Nat Genet* 51**,** 224-229.

Wang, N.-N., Li, Y., Chen, Y.-H., Lu, R., Zhou, L., Wang, Y., Zheng, Y., and Li, X.-B. (2021b). Phosphorylation of WRKY16 by MPK3-1 is essential for its transcriptional activity during fiber initiation and elongation in cotton (Gossypium hirsutum). *Plant Cell* 33**,** 2736-2752.

Wang, S., Wang, J.-W., Yu, N., Li, C.-H., Luo, B., Gou, J.-Y., Wang, L.-J., and Chen, X.-Y. (2004). Control of Plant Trichome Development by a Cotton Fiber MYB Gene. *Plant Cell* 16, 2323-2334.

Wang, X., Shen, C., Meng, P., Tan, G., and Lv, L. (2021c). Analysis and review of trichomes in plants. *BMC Plant Biol* 21**,** 1-11.

Wang, Y., Chen, W., Qin, P., Huang, Y., Ma, B., Ouyang, X., Chen, X., and Li, S. (2013). Characterization and fine mapping of Glabrous rice 2 in rice. *J Genet Genomics* 40**,** 579-582.

Wang, Y., Fu, X., Xie, L., Qin, W., Li, L., Sun, X., Xing, S., and Tang, K. (2019b). Stress associated protein 1 regulates the development of glandular trichomes in Artemisia annua. *Plant Cell, Tissue and Organ Culture* 139**,** 249-259.

Wang, Y.L., Nie, J.T., Chen, H.M., Guo, C.L., Pan, J., He, H.L., Pan, J.S., and Cai, R. (2016). Identification and mapping of Tril, a homeodomain-leucine zipper gene involved in multicellular trichome initiation in Cucumis sativus. *Theoretical and Applied Genetics* 129**,** 305-316.

Wang, Z., Wang, L., Han, L., Cheng, Z., Liu, X., Wang, S., Liu, L., Chen, J., Song, W., Zhao, J., Zhou, Z., and Zhang, X. (2021d). HECATE2 acts with GLABROUS3 and Tu to boost cytokinin biosynthesis and regulate cucumber fruit wart formation. *Plant Physiol* 187**,** 1619-1635.

Wang, Z., Yan, X., Zhang, H., Meng, Y., Pan, Y., and Cui, H. (2021e). NtCycB2 negatively regulates tobacco glandular trichome formation, exudate accumulation, and aphid resistance. *Plant Molecular Biology* 108**,** 65-76.

Wang, Z., Yang, Z., and Li, F. (2019c). Updates on molecular mechanisms in the development of branched trichome in Arabidopsis and nonbranched in cotton. *Plant Biotechnol J* 17**,** 1706-1722.

Wei, S., Gruber, M.Y., Yu, B., Gao, M.J., Khachatourians, G.G., Hegedus, D.D., Parkin, I.A., and Hannoufa, A. (2012). Arabidopsis mutant sk156 reveals complex regulation of SPL15 in a miR156-controlled gene network. *BMC Plant Biol* 12**,** 169.

Wu, M.-L., Cui, Y.-C., Ge, L., Cui, L.-P., Xu, Z.-C., Zhang, H.-Y., Wang, Z.-J., Zhou, D., Wu, S., Chen, L., and Cui, H. (2020). NbCycB2 represses Nbwo activity via a negative feedback loop in tobacco trichome development. *Journal of Experimental Botany* 71**,** 1815-1827.

Xia, X.C., Hu, Q.Q., Li, W., Chen, Y., Han, L.H., Tao, M., Wu, W.Y., Li, X.B., and Huang, G.Q. (2018). Cotton (Gossypium hirsutum) JAZ3 and SLR1 function in jasmonate and gibberellin mediated epidermal cell differentiation and elongation. *Plant Cell Tissue and Organ Culture* 133**,** 249-262.

Xiao, G., He, P., Zhao, P., Liu, H., Zhang, L., Pang, C., and Yu, J. (2018). Genome-wide identification of the GhARF gene family reveals that GhARF2 and GhARF18 are involved in cotton fibre cell initiation. *Journal of Experimental Botany* 69**,** 4323-4337.

Xie, L., Yan, T., Li, L., Chen, M., Hassani, D., Li, Y., Qin, W., Liu, H., Chen, T., Fu, X., Shen, Q., Rose, J.K.C., and Tang, K. (2021a). An HD-ZIP-MYB complex regulates glandular secretory trichome initiation in Artemisia annua. *New Phytol* 231**,** 2050-2064.

Xie, L., Yan, T., Li, L., Chen, M., Ma, Y., Hao, X., Fu, X., Shen, Q., Huang, Y., Qin, W., Liu, H., Chen, T., Hassani, D., Kayani, S.L., Rose, J.K.C., and Tang, K. (2021b). The WRKY transcription factor AaGSW2 promotes glandular trichome initiation in Artemisia annua. *J Exp Bot* 72**,** 1691-1701.

Xie, M., Sun, J., Gong, D., and Kong, Y. (2019). The Roles of Arabidopsis C1-2i Subclass of C2H2-type Zinc-Finger Transcription Factors. *Genes* 10**,** 653.

Xie, Q., Gao, Y., Li, J., Yang, Q., Qu, X., Li, H., Zhang, J., Wang, T., Ye, Z., and Yang, C. (2020a). The HD-Zip IV transcription factor SlHDZIV8 controls multicellular trichome morphology by regulating the expression of Hairless-2. *J Exp Bot* 71**,** 7132-7145.

Xie, Y.J., Yu, X.Z., Jiang, S.F., Xiao, K.Z., Wang, Y.P., Li, L.L., Wang, F.X., He, W., Cai, Q.H., Xie, H.A., and Zhang, J.F. (2020b). OsGL6, a conserved AP2 domain protein, promotes leaf trichome initiation in rice. *Biochemical and Biophysical Research Communications* 522**,** 448-455.

Xu, F., Chen, Q., Huang, L., and Luo, M. (2021). Advances about the Roles of Membranes in Cotton Fiber Development. *Membranes (Basel)* 11**,** 471.

Xu, J., Van Herwijnen, Z.O., Drager, D.B., Sui, C., Haring, M.A., and Schuurink, R.C. (2019). SlMYC1 Regulates Type VI Glandular Trichome Formation and Terpene Biosynthesis in Tomato Glandular Cells. *The Plant cell* 30**,** 2988-3005.

Xue, S.D., Dong, M.M., Liu, X.W., Xu, S., Pang, J.A., Zhang, W.Z., Weng, Y.Q., and Ren, H.Z. (2019). Classification of fruit trichomes in cucumber and effects of plant hormones on type II fruit trichome development. *Planta* 249**,** 407-416.

Xueying, G., Mingxiong, P., Gyoungju, N., Xiaoli, S., Wenxue, Y., M, S.D., and Jeffrey, C.Z. (2014). miR828 and miR858 regulate homoeologous MYB2 gene functions in Arabidopsis trichome and cotton fibre development. *Nature communications* 5**,** 3050.

Yadav, V.K., Yadav, V.K., Pant, P., Singh, S.P., Maurya, R., Sable, A., and Sawant, S.V. (2017). GhMYB1 regulates SCW stage-specific expression of the GhGDSL promoter in the fibres of Gossypium hirsutum L. *Plant Biotechnology Journal* 15**,** 1163-1174.

Yan, A., Wu, M., Zhao, Y., Zhang, A., Liu, B., Schiefelbein, J., and Gan, Y. (2014). Involvement of C2H2 zinc finger proteins in the regulation of epidermal cell fate determination in Arabidopsis. *J Integr Plant Biol* 56**,** 1112-1117.

Yan, T., Chen, M., Shen, Q., Li, L., Fu, X., Pan, Q., Tang, Y., Shi, P., Lv, Z., and Jiang, W. (2017). HOMEODOMAIN PROTEIN 1 is required for jasmonate‐mediated glandular trichome initiation in Artemisia annua. *New Phytol* 213**,** 1145-1155.

Yan, T., Li, L., Xie, L., Chen, M., Shen, Q., Pan, Q., Fu, X., Shi, P., Tang, Y., Huang, H., Huang, Y., Huang, Y., and Tang, K. (2018). A novel HD‐ZIP IV/MIXTA complex promotes glandular trichome initiation and cuticle development in Artemisia annua. *New Phytol* 218, 567-578.

Yang, C., Gao, Y., Gao, S., Yu, G., Xiong, C., Chang, J., Li, H., and Ye, Z. (2015). Transcriptome profile analysis of cell proliferation molecular processes during multicellular trichome formation induced by tomato Wov gene in tobacco. *BMC Genomics* 16**,** 868.

Yang, S., Cai, Y., Liu, X., Dong, M., Zhang, Y., Chen, S., Zhang, W., Li, Y., Tang, M., and Zhai, X. (2018). A CsMYB6-CsTRY module regulates fruit trichome initiation in cucumber. *J Exp Bot* 69**,** 1887-1902.

Yang, X., Zhang, W., He, H., Nie, J., Bie, B., Zhao, J., Ren, G., Li, Y., Zhang, D., Pan, J., and Cai, R. (2014a). Tuberculate fruit gene Tu encodes a C2 H2 zinc finger protein that is required for the warty fruit phenotype in cucumber (Cucumis sativus L.). *Plant J* 78**,** 1034-1046.

Yang, Z., Song, M., Cheng, F., Zhang, M., Davoudi, M., Chen, J., and Lou, Q. (2021). A SNP Mutation in Homeodomain-DDT (HD-DDT) Transcription Factor Results in Multiple Trichomes (mt) in Cucumber (Cucumis sativus L.). *Genes (Basel)* 12**,** 1478.

Yang, Z., Zhang, C., Yang, X., Liu, K., Wu, Z., Zhang, X., Zheng, W., Xun, Q., Liu, C., and Lu, L. (2014b). PAG1, a cotton brassinosteroid catabolism gene, modulates fiber elongation. *Genes (Basel)* 203**,** 437-448.

Yang, Z.B. (2002). Small GTPases: Versatile signaling switches in plants. *Plant Cell* 14**,** S375-S388.

Yanna, G., Shenghua, G., Cheng, X., Gang, Y., Jiang, C., Zhibiao, Y., and Changxian, Y. (2015). Comprehensive analysis and expression profile of the homeodomain leucine zipper IV transcription factor family in tomato. *Plant physiology and biochemistry : PPB* 96**,** 141-153.

Yong-Mei, Q., and Yu-Xian, Z. (2011). How cotton fibers elongate: a tale of linear cell-growth mode. *Curr Opin Plant Biol* 14**,** 106-111.

Yu, D., Li, X., Li, Y., Ali, F., Li, F., and Wang, Z. (2021). Dynamic roles and intricate mechanisms of ethylene in epidermal hair development in Arabidopsis and cotton. *New Phytol*. 234**,** 375-391.

Yu, N., Cai, W.J., Wang, S., Shan, C.M., Wang, L.J., and Chen, X.Y. (2010). Temporal control of trichome distribution by microRNA156-targeted SPL genes in Arabidopsis thaliana. *Plant Cell* 22**,** 2322-2335.

Yuan, D., Tang, Z., Wang, M., Gao, W., Tu, L., Jin, X., Chen, L., He, Y., Zhang, L., Zhu, L., Li, Y., Liang, Q., Lin, Z., Yang, X., Liu, N., Jin, S., Lei, Y., Ding, Y., Li, G., Ruan, X., Ruan, Y., and Zhang, X. (2015). The genome sequence of Sea-Island cotton (Gossypium barbadense) provides insights into the allopolyploidization and development of superior spinnable fibres. *Sci Rep* 5**,** 17662.

Zhang, A., Liu, Y., Yu, C., Huang, L., Wu, M., Wu, J., and Gan, Y. (2020). Zinc Finger Protein 1 (ZFP1) Is Involved in Trichome Initiation in Arabidopsis thaliana. *Agriculture-Basel* 10**,** 645.

Zhang, F., Zuo, K., Zhang, J., Liu, X., Zhang, L., Sun, X., and Tang, K. (2010). An L1 box binding protein, GbML1, interacts with GbMYB25 to control cotton fibre development. *Journal of Experimental Botany* 61**,** 3599-3613.

Zhang, H., Li, W., Niu, D., Wang, Z., Yan, X., Yang, X., Yang, Y., and Cui, H. (2019). Tobacco transcription repressors NtJAZ: Potential involvement in abiotic stress response and glandular trichome induction. *Plant Physiol Biochem* 141**,** 388-397.

Zhang, J., Huang, G.Q., Zou, D., Yan, J.Q., Li, Y., Hu, S., and Li, X.B. (2018). The cotton (Gossypium hirsutum) NAC transcription factor (FSN1) as a positive regulator participates in controlling secondary cell wall biosynthesis and modification of fibers. *New Phytol* 217**,** 625-640.

Zhang, M., Xiao, Y., Zeng, J., and Pei, Y. (2017). PIN-formed protein, a door to reveal the mechanism for auxin-triggered initiation of cotton fiber. *Plant Signaling & Behavior* 12**,** e1319031.

Zhang, X., Cao, J., Huang, C., Zheng, Z., Liu, X., Shangguan, X., Wang, L., Zhang, Y., and Chen, Z. (2021a). Characterization of cotton ARF factors and the role of GhARF2b in fiber development. *Bmc Genomics* 22**,** 202.

Zhang, X., Xue, Y., Guan, Z., Zhou, C., Nie, Y., Men, S., Wang, Q., Shen, C., Zhang, D., and Jin, S. (2021b). Structural insights into homotrimeric assembly of cellulose synthase CesA7 from Gossypium hirsutum. *Plant Biotechnol J* 19**,** 1579-1587.

Zhang, Y., Shen, J., Bartholomew, E.S., Dong, M., Chen, S., Yin, S., Zhai, X., Feng, Z., Ren, H., and Liu, X. (2021c). TINY BRANCHED HAIR functions in multicellular trichome development through an ethylene pathway in Cucumis sativus L. *Plant J* 106**,** 753-765.

Zhang, Z., Chen, X., Guan, X., Liu, Y., Chen, H., Wang, T., Mouekouba, L.D.O., Li, J., and Wang, A. (2014). A genome-wide survey of homeodomain-leucine zipper genes and analysis of cold-responsive HD-Zip I members’ expression in tomato. *Biosci Biotechnol Biochem* 78**,** 1337-1349.

Zhao, J.-L., Pan, J.-S., Guan, Y., Zhang, W.-W., Bie, B.-B., Wang, Y.-L., He, H.-L., Lian, H.-L., and Cai, R. (2015). Micro-trichome as a class I homeodomain-leucine zipper gene regulates multicellular trichome development in Cucumis sativus. *Journal of Integrative Plant Biology* 57**,** 925-935.

Zhao, T., Tao, X., Li, M., Gao, M., Chen, J., Zhou, N., Mei, G., Fang, L., Ding, L., and Zhou, B. (2020). Role of phasiRNAs from two distinct phasing frames of GhMYB2 loci in cis-gene regulation in the cotton genome. *BMC Plant Biol* 20**,** 1-13.

Zheng, F., Cui, L., Li, C., Xie, Q., Ai, G., Wang, J., Yu, H., Wang, T., Zhang, J., Ye, Z., and Yang, C. (2021). Hair (H) interacts with SlZFP8-like to regulate the initiation and elongation of trichomes by modulating SlZFP6 expression in tomato. *J Exp Bot*. 73**,** 228-244.

Zhou, Y., Li, B.-Y., Li, M., Li, X.-J., Zhang, Z.-T., Li, Y., and Li, X.-B. (2014). A MADS-box gene is specifically expressed in fibers of cotton (Gossypium hirsutum) and influences plant growth of transgenic Arabidopsis in a GA-dependent manner. *Plant Physiol Biochem* 75**,** 70-79.

Zhou, Z., An, L., Sun, L., and Gan, Y. (2012). ZFP5 encodes a functionally equivalent GIS protein to control trichome initiation. *Plant signaling & behavior* 7**,** 28-30.

Zhou, Z., An, L., Sun, L., Zhu, S., Xi, W., Broun, P., Yu, H., and Gan, Y. (2011). Zinc Finger Protein5 Is Required for the Control of Trichome Initiation by Acting Upstream of Zinc Finger Protein8 in Arabidopsis. *Plant Physiology* 157**,** 673-682.

Zhou, Z., Sun, L., Zhao, Y., An, L., Yan, A., Meng, X., and Gan, Y. (2013). Zinc Finger Protein 6 (ZFP6) regulates trichome initiation by integrating gibberellin and cytokinin signaling in Arabidopsis thaliana. *New Phytologist* 198**,** 699-708.

Zhou, Z., Tan, H.X., Li, Q., Li, Q., Wang, Y., Bu, Q.T., Li, Y.X., Wu, Y., Chen, W.S., and Zhang, L. (2020). TRICHOME AND ARTEMISININ REGULATOR 2positively regulates trichome development and artemisinin biosynthesis inArtemisia annua. *New Phytologist* 228, 932-945.
