# Supplementary material for: Molecular Mechanisms of Plant Trichome Development
Source: Front Plant Sci. 2022 Jun 1;13:910228. doi: 10.3389/fpls.2022.910228 (PMC9198495; doi:10.3389/fpls.2022.910228)
Supplement: Supplementary file 2 [file Data_Sheet_1.docx]

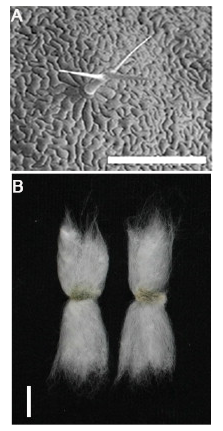


Supplementary Figure 1 Morphology of unicellular trichomes (Breuer et al., 2009;Guan et al., 2014) .

(A) SEM observation of *Arabidopsis* trichomes, 10-day-old wild-type (Columbia [Col]) first true leaf. Scale bar = 400 μm; (B) Fibers on cottonseed of upland cotton (*Cossypium hirsutum* L.acc.TM-1). Phytogen 800. Scale bar of = 1 cm.


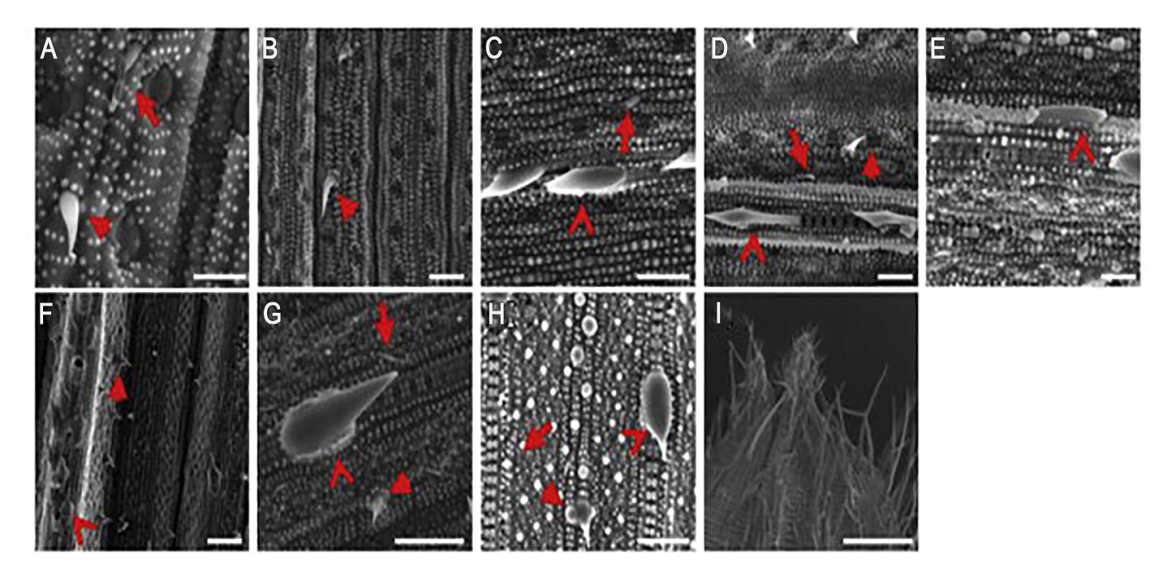


Supplementary Figure 2 Microscopic observation of trichomes of wild-type rice (Wang et al., 2013).

(A) The adaxial surface at the first leaf stage; (B) Leaf adaxial surface at the third leaf stage; (C) Leaf abaxial surface at the third leaf stage; (D) Leaf adaxial surface at the sixth leaf stage; (E) Leaf adaxial surface at the sixth leaf stage; (F) Leaf adaxial surface at the flowering stage; (G) A higher magnification of leaf adaxial surface was observed during the flowering stage (in F); (H) Leaf abaxial surface at the flowering stage; (I) the tip of rice hull. Scale bars = 40 μm in A, B, F, H and I; 30 μm in C, D, E, and G. Arrow: glandular hair; arrow head: microhair; empty arrow head: macrohair.


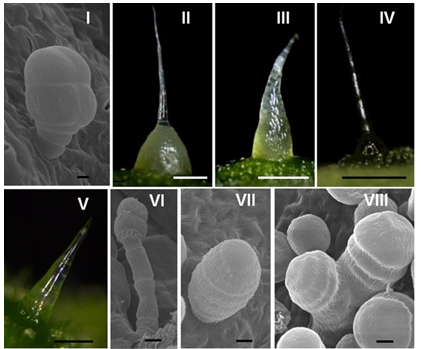


Supplementary Figure 3 Morphological characteristics of eight (I–VIII) types of trichomes on cucumber fruits. Scale bar =  500 μm (II-IV); 100 μm (V); 20 μm (VI); and 5 μm (I, VII and VIII) (Xue et al., 2019).


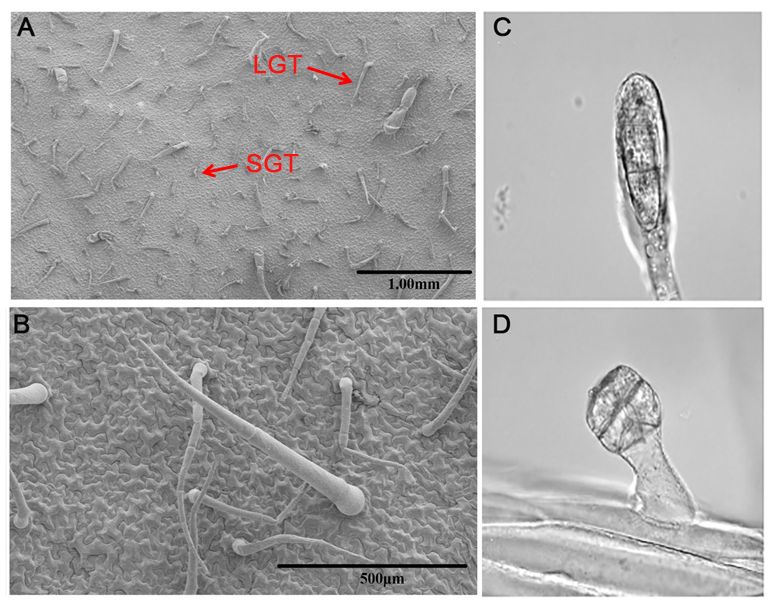


Supplementary Figure 4 Glandular trichomes of tobacco (Amme et al., 2005;Liu et al., 2018).

(A) Scanning electron microscopy analysis of long-stalked glandular trichomes (LGT), and short-stalked glandular trichomes (SGT) in wild-type leaves. Scale bar = 1.00 mm; (B) Scanning electron microscopy analysis of LGT in wild-type leaves. Scale bar = 500 μm; (C) Multicellular stem and head of LGT; (D) Unicellular stem and multicellular head of SGT.


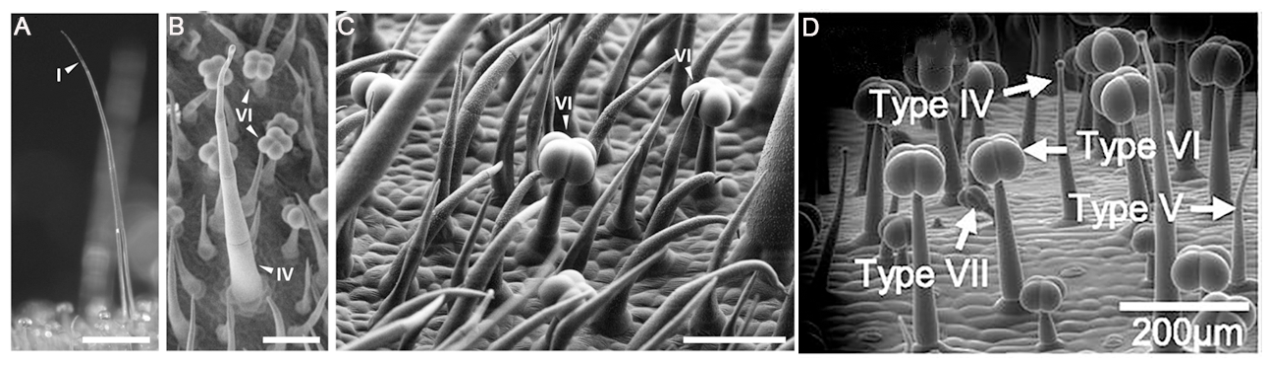


Supplementary Figure 5 Different types of trichomes of tomato (Xu et al., 2018;Chalvin et al., 2020).

(A) Type I trichome of a tomato stem observed with a zoom stereomicroscope. Scale bar = 500 μm; (B) Type IV and VI trichomes of a tomato leaf (adaxial face) observed with a scanning electron microscope. Scale bars = 100 μm; (C) Type IV and VI trichomes of a tomato leaf (adaxial face) observed with a scanning electron microscope. Scale bars = 100 μm; (D) Type IV, V, VI and VII trichomes of wild-type tomato stem surface. Scale bars = 200 μm.


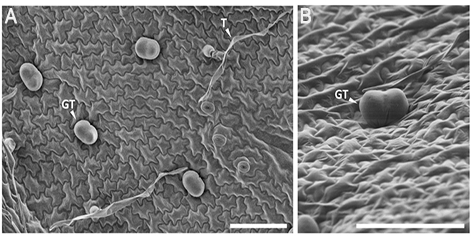


Supplementary Figure 6 Glandular trichomes of *Artemisia annua* (Chalvin et al., 2020).

(A) The trichomes of *A. annua* leaf (adverse) were observed by scanning electron microscope. Scale bar = 100 μm; (B) The trichomes of *A. annua* leaf (adverse) were observed by scanning electron microscope. Scale bar = 100 μm.

**References**

Amme, S., Rutten, T., Melzer, M., Sonsmann, G., Vissers, J.P.C., Schlesier, B., and Mock, H.P. (2005). A proteome approach defines protective functions of tobacco leaf trichomes. *Proteomics* 5**,** 2508-2518.

Breuer, C., Kawamura, A., Ichikawa, T., Tominaga-Wada, R., Wada, T., Kondou, Y., Muto, S., Matsui, M., and Sugimoto, K. (2009). The trihelix transcription factor GTL1 regulates ploidy-dependent cell growth in the Arabidopsis trichome. *Plant Cell* 21**,** 2307-2322.

Chalvin, C., Drevensek, S., Dron, M., Bendahmane, A., and Boualem, A. (2020). Genetic Control of Glandular Trichome Development. *Trends Plant Sci* 25**,** 477-487.

Guan, X., Song, Q., and Chen, Z.J. (2014). Polyploidy and small RNA regulation of cotton fiber development. *Trends Plant Sci* 19**,** 516-528.

Liu, Y., Liu, D., Khan, A.R., Liu, B., Wu, M., Huang, L., Wu, J., Song, G., Ni, H., Ying, H., Yu, H., and Gan, Y. (2018). NbGIS regulates glandular trichome initiation through GA signaling in tobacco. *Plant Mol Biol* 98**,** 153-167.

Wang, Y., Chen, W., Qin, P., Huang, Y., Ma, B., Ouyang, X., Chen, X., and Li, S. (2013). Characterization and fine mapping of Glabrous rice 2 in rice. *J Genet Genomics* 40**,** 579-582.

Xu, J., Van Herwijnen, Z.O., Dräger, D.B., Sui, C., Haring, M.A., and Schuurink, R.C. (2018). SlMYC1 Regulates Type VI Glandular Trichome Formation and Terpene Biosynthesis in Tomato Glandular Cells. *Plant Cell* 30**,** 2988-3005.

Xue, S., Dong, M., Liu, X., Xu, S., Pang, J., Zhang, W., Weng, Y., and Ren, H. (2019). Classification of fruit trichomes in cucumber and effects of plant hormones on type II fruit trichome development. *Planta* 249**,** 407-416.
